# Exploring the Role of Advanced MRI in Understanding Glioblastoma Biology: A Scoping Review

**DOI:** 10.3390/cancers18040645

**Published:** 2026-02-16

**Authors:** James Brown-Miles, Oun Al-Iedani, Hubert Hondermarck, Peter Greer, Michael Fay, Saadallah Ramadan

**Affiliations:** 1 School of Biomedical Sciences and Pharmacy, College of Health, Medicine and Wellbeing, University of Newcastle, Newcastle, NSW 2308, Australia; james.brownmiles@uon.edu.au (J.B.-M.); oun.aliedani@newcastle.edu.au (O.A.-I.); 2 Hunter Medical Research Institute, Newcastle, NSW 2305, Australia; peter.greer@newcastle.edu.au (P.G.); michael.fay@newcastle.edu.au (M.F.); 3 Mark Hughes Foundation Centre for Brain Cancer Research, University of Newcastle, Newcastle, NSW 2305, Australia; 4 NSW Regional Cancer Research Network, NSW Regional Health Partners, Cancer Institute NSW, Newcastle, NSW 2305, Australia; 5 School of Information and Physical Sciences, College of Engineering, Science and Environment, University of Newcastle, Newcastle, NSW 2308, Australia; 6 Department of Radiation-Oncology, Calvary Mater Newcastle, Newcastle, NSW 2298, Australia; 7 School of Medicine and Public Health, College of Health, Medicine and Wellbeing, University of Newcastle, Newcastle, NSW 2308, Australia; 8 GenesisCare, Lake Macquarie Private Hospital, Newcastle, NSW 2290, Australia

**Keywords:** magnetic resonance imaging (MRI), advanced MRI, multiparametric imaging, glioblastoma (GBM), preoperative, brain cancer, neuro-oncology, brain tumour biology

## Abstract

Background: Among adult primary brain tumours, glioblastoma (GBM) carries the worst prognosis. Magnetic resonance imaging (MRI) serves to diagnose and guide treatment, despite recognised constraints. Advanced MRI techniques—magnetic resonance spectroscopy (MRS), amide proton transfer-weighted imaging (APTw), diffusion-weighted imaging (DWI), perfusion-weighted imaging (PWI), and quantitative susceptibility mapping (QSM)—reveal GBM characteristics that conventional sequences cannot detect. The 2021 World Health Organization reclassification disrupted established imaging–biology relationships, necessitating isocitrate dehydrogenase wildtype (IDHwt) GBM-specific evidence integration. This review synthesises biological insights provided by advanced MRI in preoperative IDHwt GBM. Methods: We conducted a scoping review following the PRISMA-ScR framework, querying five databases (PubMed, Scopus, Cochrane, EBSCO, and Embase) to identify literature using advanced MRI to investigate biological correlates in IDHwt GBM. Results: MRS ratios were associated with tumour presence and Ki-67 expression. APTw demonstrated robust associations with cellularity but failed to predict O6-methylguanine-DNA methyltransferase promoter (MGMTp) methylation. DWI exhibited variable utility; certain metrics linked to cellularity, while apparent diffusion coefficient values were inconsistent but useful for predicting MGMTp status, telomerase reverse transcriptase promoter mutations, and CD163+ macrophage infiltration. PWI showed relationships with cellularity, micro-vessel area, and MGMTp status. QSM features correlated with Ki-67, ferritin, and immune markers. Conclusions: Advanced MRI demonstrates potential for biological stratification of GBM, though protocol variability and limited reproducibility hinder clinical translation. Standardised pipelines and prospective multicentre validation must precede clinical adoption, before these techniques can benefit patients.

## Simple Summary

This review brings together past imaging research and aligns it with the 2021 World Health Organization classification to provide a clearer picture of glioblastoma biology. Glioblastoma is the deadliest brain tumour in adults, and traditional imaging often fails to reveal its complex biology. We explore how advanced magnetic resonance imaging techniques can uncover features such as cell growth, blood supply, and immune response without invasive procedures. For example, amide proton transfer-weighted imaging detects elevated protein and peptide content in highly proliferative tumour regions, correlating with the histological proliferation marker Ki-67. By identifying the most promising methods and highlighting gaps in knowledge, this work aims to guide future studies. If adopted clinically, these techniques could improve diagnosis, enable personalised treatment, and help predict outcomes, ultimately advancing care for people with glioblastoma.

## 1. Introduction

### 1.1. Rationale

Magnetic resonance imaging (MRI) represents a fundamental tool for glioblastoma (GBM) assessment in both clinical practice and research [1]. Conventional MRI exhibits well-documented constraints. T1-weighted (T1w) gadolinium-enhanced sequences cannot detect microscopic tumour infiltration extending beyond the primary lesion, as contrast enhancement (CE) reflects where the blood–brain barrier is compromised rather than cellular invasion [2]. This limitation has significant implications for the outcomes of treatment, since conventional MRI is used for surgical planning, guiding radiotherapy, and monitoring for progression. It necessitates the development and validation of advanced MRI methods—such as diffusion-weighted imaging (DWI), perfusion-weighted imaging (PWI), magnetic resonance spectroscopy (MRS), chemical exchange saturation transfer (CEST), and susceptibility-based methods—for the improved diagnosis and prognostication of GBM. Many researchers have investigated the correlation between particular magnetic resonance (MR) metrics and specific biological factors [3,4,5,6], suggesting potential for the non-invasive characterisation of GBM.

GBM is notoriously heterogeneous, and in 2021, the World Health Organization (WHO) classification was updated to incorporate histological and molecular criteria [7]. This revision separated ‘glioblastoma multiforme’ into two distinct tumour types: WHO grade 4 GBM isocitrate dehydrogenase wildtype (IDHwt) and WHO grade 4 astrocytoma, IDH-mutant (IDHmt), thereby necessitating reinterpretation of historical imaging literature. While the IDH mutation status is a key molecular marker, it is not the only one to play a significant prognostic role in the management of GBM [8]. Other key factors include the O6-methylguanine-DNA methyltransferase promoter (MGMTp) methylation status [9,10], epidermal growth factor receptor (EGFR) amplification status [11], and telomerase reverse transcriptase promoter (TERTp) mutations [12], which also impacts treatment response and prognosis. Numerous exploratory studies have investigated the potential correlation between advanced MRI metrics and relevant biological factors [3,4,5,6], suggesting opportunities for the non-invasive characterisation of GBM. Herein, GBM will be used in line with the 2021 classification.

No synthesis has yet comprehensively mapped advanced MRI correlates of biological mechanisms in GBM, particularly across molecular subtypes. Prior reviews [13,14,15,16,17] focused on the technical imaging parameters, specific applications of deep-learning models, or general biomarker identification without molecular stratification or alignment with contemporary WHO classification criteria, resulting in limited clinical applicability. Hence, the goal of the present review is to harmonise the field’s existing research in accordance with the 2021 WHO reclassification, to provide a comprehensive characterisation of a more homogeneous cohort of adult, preoperative, IDHwt GBM patients. This is possible by the approximate equivalence of historical primary/de novo GBM to the 2021 WHO classification of GBM IDHwt, while the historical secondary GBM equates to grade 4 IDHmt gliomas [18,19,20,21]. The goal of this review is to reveal how radiologic interpretations can confer a better understanding of differences between molecular subtypes of GBM IDHwt. In this review, we aim to highlight the current state of knowledge to identify any gaps in the literature and inform the direction of future research.

#### 1.1.1. Molecular Markers

Methylation of MGMTp epigenetically silences MGMT, enhancing alkylating agent susceptibility (e.g., temozolomide [TMZ], the standard of care first-line chemotherapy), which confers a corresponding survival benefit [22,23,24].

Amplification of EGFR occurs commonly in GBM and constitutes, with TERTp mutations, a key 2021 WHO diagnostic molecular criterion reflecting oncogenic pathway activation driving aggressive growth [7,25,26,27].

TERTp mutations drive telomerase upregulation and associate with infiltrative patterns on DWI, particularly reduced ADC in non-enhancing/FLAIR-hyperintense zones, underscoring diagnostic importance [26,27].

#### 1.1.2. MR Modalities

Advanced MRI techniques measure unique properties; DWI quantifies the movement of water, characterising microstructural alterations (including cellular density), within the tumour and surrounding parenchyma [28]. Diffusion tensor imaging (DTI) is an extension of DWI, which detects subtle disruptions in white matter tracts [29].

PWI evaluates tumour haemodynamics, typically the flow and volume of cerebral blood, contributing to diagnostic assessment [1].

MRS detects chemical shift variations in endogenous metabolite hydrogens, yielding concentration measurements [30]. Meanwhile, amide proton transfer-weighted (APTw) imaging applies CEST principles to assess amide proton exchange between endogenous mobile proteins/peptides and bulk water, indirectly reflecting protein levels and pH [31].

Susceptibility-weighted imaging (SWI) and quantitative susceptibility mapping (QSM) detect iron deposition and calcification via local magnetic field distortion quantification [32].

### 1.2. Objectives

This scoping review was performed in line with a prespecified protocol [33] to survey the existing knowledge that combines radiology, neuro-oncology, and biology. The research question underpinning this scoping review was “Can advanced MRI help understand the biology of GBM?” To address this, the following five research questions (RQs) were developed:

- RQ1: What biological insights have been established using advanced MRI for GBM?
- RQ2: Which advanced MRI modality shows the most utility, determined by their correlation with, or ability to predict the status of, histological and molecular markers, for elucidating biological insights into GBM, and what study-level acquisition/processing choices influence this utility?
- RQ3: Which advanced MRI technologies are currently under-researched and/or under-utilised for investigating GBM-related biology?
- RQ4: What aspects of GBM-related biology are currently under-explored using advanced MRI techniques?
- RQ5: What are the typical limitations of studies using advanced MRI to investigate GBM-related biology?

## 2. Materials and Methods

### 2.1. Protocol and Registration

This scoping review followed a prespecified protocol [33], Preferred Reporting Items for Systematic Reviews and Meta-Analyses extension for Scoping Reviews (PRISMA-ScR) reporting standards [34], and the Joanna Briggs Institute (JBI) methodological recommendations for scoping reviews [35,36,37]. While an a priori protocol was developed [33] and has undergone peer review before implementation, it was not registered in a public registry such as the Open Science Framework (https://osf.io/, accessed on 26 January 2026). We acknowledge that not registering it publicly reduces transparency relative to studies with publicly registered protocols, as is recommended by current reporting guidelines [34]. A scoping review was deemed most appropriate to map how advanced MRI has been used to interrogate GBM biology across diverse study designs, image-acquisition strategies, and to identify where standardisation is sufficient to support future quantitative synthesis. Scoping reviews are specifically recommended when the literature is broad and methodologically diverse, and when the aim is to characterise concepts, methods, and gaps rather than to estimate a single pooled effect, synthesise effect sizes, or assess intervention efficacy [34,38]. In contrast, systematic reviews and meta-analyses presuppose a narrow question and comparable effect measures [34,38]. Our scoping review differs from past reviews as it is anchored to the 2021 WHO adult-type diffuse glioma taxonomy, whereas earlier reviews often aggregated mixed IDH cohorts or tumour grades and have not systematically mapped the relationships between advanced MRI techniques and the underlying biological processes that drive GBM behaviour. Herein, a summary of the protocol [33], and details of any deviations made are provided.

### 2.2. Eligibility Criteria

Inclusion criteria prioritised advanced MRI utility for preoperative GBM characterisation. The full criteria are detailed in the protocol [33], a brief description of the key criteria is provided in Table 1. Per protocol specifications, investigations solely presenting machine learning (ML), artificial intelligence (AI), or radiomics-derived metrics were deemed ineligible. This criterion was to both bound the scope of the review and to maintain a biology-focused synthesis. Future reviews may focus on these approaches, which are an evolving field deserving of a dedicated review.

### 2.3. Information Sources

As per the protocol [33], we systematically searched PubMed, Scopus, Cochrane, Ebsco (Academic Search Ultimate), and Embase (Ovid) for primary sources of evidence that comply with the eligibility criteria of this scoping review. These database searches were not supplemented by reference-list-scanning or hand-searching of key journals.

### 2.4. Search Strategy

The search strategy entered into each of the five databases was comprising the keywords (and variations for to capture each MRI modality and the heterogenous vocabulary to describe them) “(MRI OR “magnetic resonance” OR “APT” OR “CEST” OR “DWI” OR “DTI” OR “SWI” OR “QSM” OR “DKI” OR “PWI”) AND (glioblastoma OR GBM OR “high-grade glioma”).” Further details on the search strategy are available in the protocol [33] and complete details regarding the search terms, search limitations, and filters applied for each database are listed in Supplementary Materials Table S1.

This search strategy was implemented on 10 September 2025, and automated alerts were set up to continuously monitor for newly published articles that match the initial search strategy. These were screened according to the same eligibility criteria; the last update screened for inclusion in this review was received on 18 December 2025.

### 2.5. Selection of Sources of Evidence

In accordance with the protocol [33], study screening and selection were managed in Covidence (Covidence systematic review software, Veritas Health Innovation, Melbourne, Australia. Available at www.covidence.org, accessed on 18 December 2025) [39], which supports collaborative screening and automated removal of duplicate records. Dual reviewers independently executed sequential ‘Title and abstract screening’ and ‘Full text review’. Discrepancies underwent consensus adjudication, with arbitration by a third reviewer when necessary. Inter-reviewer agreement was quantified using Cohen’s κ, reporting the level of agreement alongside the percent agreement [34,40,41].

### 2.6. Data Charting Process

In line with the protocol [33], the results that passed the screening process were exported to EndNote (Clarivate, Philadelphia, PA, USA) for manual data extraction. A single reviewer conducted data charting utilising a data extraction matrix created in Microsoft Word (Microsoft Corporation, Redmond, WA, USA; Supplementary Materials Table S2). A second reviewer independently verified the data extraction process for a subset (approximately 30%) of eligible records to verify accuracy and reduce bias, while balancing this quality control with feasibility.

This review operated under the premise that pre-2021 investigations of de novo/primary GBM without specified IDH status align with the contemporary GBM IDHwt classification, permitting incorporation of historical findings. Meanwhile, ‘secondary GBM’ was excluded from this review, as it equates to WHO grade 4 astrocytoma, IDHmt, which is not the focus of the present review. This assumption and its impact are further discussed in the protocol [33].

### 2.7. Synthesis of Results

Included studies were synthesised according to a structured framework that groups them by the biological domain (molecular factors, microenvironmental, vascular, metabolic, immune response) they report on, with subgroups defined by the MRI modalities (or combination of such) that they utilised. Within these groups, relevant MRI-derived metrics and key findings were summarised, with additional notes made regarding the sample size of the study cohort, the aim of the research, and whether the IDHwt status was reported or inferred. Further details are provided in the protocol [33].

## 3. Results

### 3.1. Characteristics of Sources of Evidence

**Figure 1:**
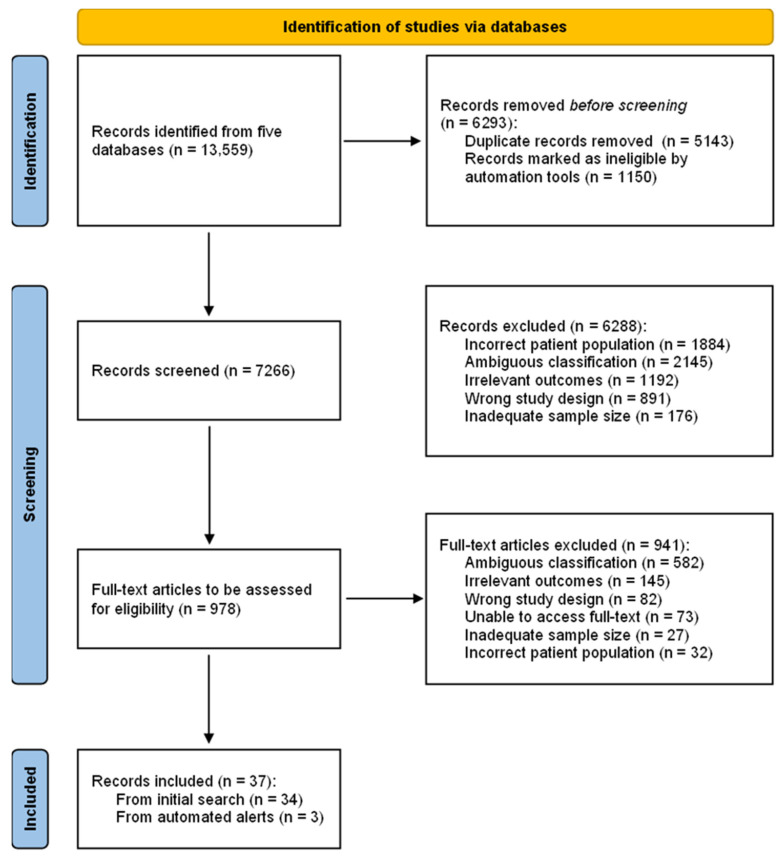
The PRISMA flow diagram depicts the number of records throughout each step of the scoping review, from identification to the screening of studies and final inclusion.

**Figure 2:**
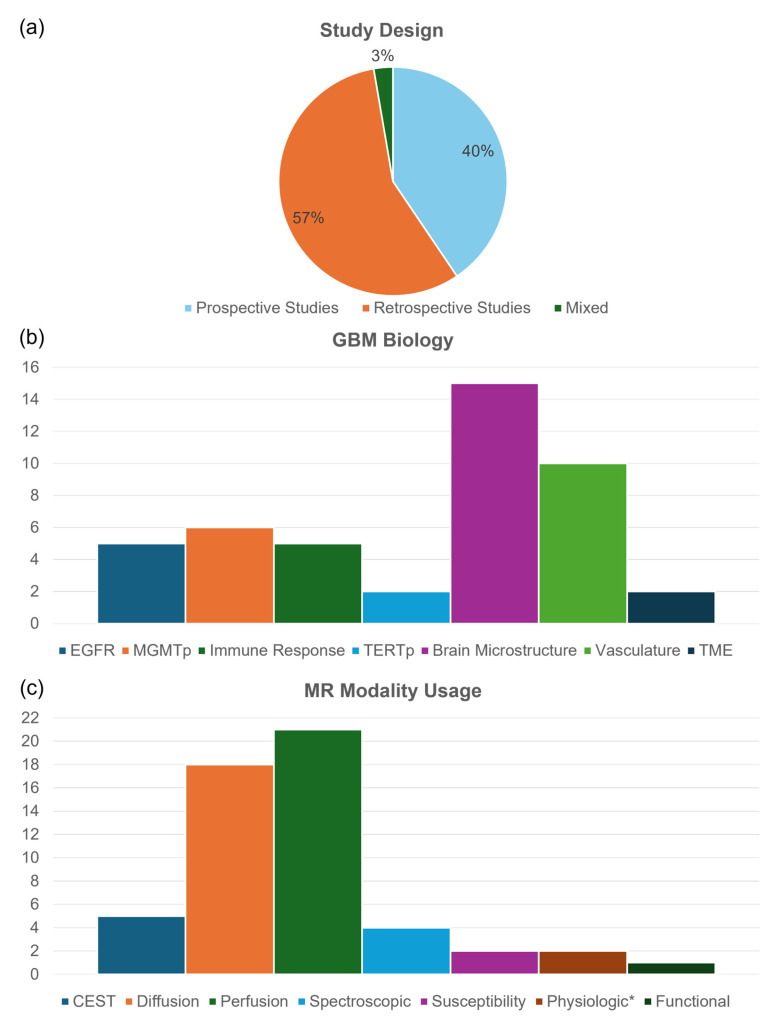
The characteristics of the included records. (**a**) The distribution of study designs. (**b**) The frequency with which each biological aspect was investigated. (**c**) The frequency with which each MR modality was utilised. *Physiologic MRI included quantitative blood oxygen level-dependent (BOLD) oxygen-metabolism mapping with vascular architecture mapping. Note: The sum of frequencies in panels (**b**,**c**) exceeds 37 because categories are not mutually exclusive; a single reference may be counted in multiple categories. Abbreviations: GBM, glioblastoma; EGFR, epidermal growth factor receptor; MGMTp, O6-methylguanine-DNA methyltransferase promoter; TERTp, telomerase reverse transcriptase promoter; TME, tumour microenvironment; MR, magnetic resonance; CEST, chemical exchange saturation transfer.

Database searches yielded 13,559 records (Figure 1 PRISMA flow diagram), and 37 studies were eligible for inclusion in this review. The distribution of biological aspects investigated and the MRI modalities utilised are shown in Figure 2. The characteristics of individual records are shown in Table 2.

Title/abstract screening inter-reviewer agreement reached 86% of observed agreement (Cohen’s κ = 0.78), increasing to 92% of observed agreement (Cohen’s κ = 0.84) during full-text screening.

### 3.2. Results of Individual Sources of Evidence

#### 3.2.1. Brain Microstructure: Proliferation and Invasion

Table 3 summarises findings from the 15 studies investigating GBM microstructure.

Inoue et al. [53] assessed APTw imaging’s clinical utility for malignant glioma metabolic characterisation, comparing findings with both immunohistochemistry (IHC) and carbon-11-labelled methionine positron emission tomography (11C-MET-PET) analyses. Regions of interest (ROIs) were placed either in the CE tumour or in the absence of CE in areas of abnormal signal on fluid attenuated inversion recovery (FLAIR) images. The average Ki-67 labelling index (LI) was 37% ± 14, and the average APT_mean_ for all ROIs was 27.2% ± 12.8, which was higher than in other gliomas (*p* < 0.001). Inoue et al. concluded that APTw imaging may provide the exact location of infiltrating tumour cells. Ohba et al. [55] evaluated whether APTw imaging is useful for distinguishing primary central nervous system lymphoma (PCNSL) from GBM. Ohba et al. reported a moderate correlation between mean APTw signal and MIB-1 LI; however, APTw percentiles showed no association, indicating mean values may better reflect proliferative potential in GBM. They also assessed whether there were correlations between a predictable diagnosis of GBM and p53 immunopositivity. Ohba et al. found no correlation between the diagnosis predicted by APTw imaging and p53 immunopositivity, or significant differences in the mean, 1st and 100th percentile, or width1–100 APTw signals between p53-positive and p53-negative GBMs. Yuan et al. [63] combined APTw and MRS to explore correlations between the two modalities before investigating the potential to apply multi-modal MRI for metabolic insights and tumour border delineation, with reference to Fluoroethyl-tyrosine-PET (FET). They revealed that APTw imaging and the MRS-derived choline (Cho) to N-acetylaspartate (NAA) ratio (CNR) were strongly correlated with FET values at the voxel-wise level. Additionally, voxel-wise analysis showed a moderate correlation between CEST and MRS; however, patient-wise analysis revealed no CEST-MRS correlation. Probability maps derived from CEST and MRS combined were reported to have the best efficacy in predicting the presence of the tumour, thereby confirming that CEST and MRS can evaluate the metabolic activity of gliomas, be it through different mechanisms. Schon et al. [68] utilised CEST-APT and dynamic susceptibility contrast (DSC)-PWI data to investigate the relationship between these imaging metrics and the cellularity in newly diagnosed gliomas. They report a robust association between cellularity and APTw signals (Spearman’s ρ = 0.37, *p* = 0.02886). However, cerebral blood flow (CBF) did not correlate with cellularity (Spearman’s ρ = 0.11, *p* = 0.52). Findings suggested there is a potential synergism in the combination of APTw and cerebral blood volume (CBV) information. Blasel et al. [76] employed DSC and 2D-MR spectroscopic imaging (MRSI) to characterise the distinct striate sign (SS), which they hypothesised precedes future CE tumour. The SS was defined as an area of relative CBV (rCBV) increase adjacent to the CE tumour that extends from the border in a defined direction. The SS of all patients (n = 6) that showed new CE at follow-up (FU) (nine months post-treatment) had increased total Cho (tCho) concentrations at baseline. Blasel et al. suggest that the SS may be a marker of directed tumour cell infiltration that is capable of predicting the location and direction of GBM recurrence. Valentini et al. [72] aimed to identify, in vivo, the most malignant regions of the tumour, the extent of infiltration, and the sites of glioma stem cell (GSC) origination by using a combination of imaging modalities, including MRS, DWI, PWI, and fluorine-18 fluorodeoxyglucose PET (18F-FDG)/computed tomography (CT). They collected biopsy specimens that correspond to radiologically defined tumour regions. Valentini et al. report that the Ki-67/MIB-1 LI was associated with the Cho/creatine (Cho/Cr) values (r = 0.95, *p* = 0.03). Furthermore, the rCBV, Cho/Cr, Cho/NAA, and maximum 18F-FDG standardised uptake value (SUV_max_) values in the CE regions were shown to provide the best evaluation of tumour malignancy and were reflective of the areas with the highest Ki-67/MIB-1 LI.

Wurtemberger et al. [51] employed multi-shell DWI to noninvasively delineate GBM microarchitecture. In their analysis, GBM displayed a marked reduction in anisotropy and volume fraction metrics (intracellular volume fraction [ICVF], intra-neurite volume fraction [V-intra], fractional anisotropy [FA], and microscopic fractional anisotropy [microFA]) relative to normal-appearing white matter (NAWM). Meanwhile, mean diffusivity (MD), radial diffusivity (RD), axial diffusivity (AD), orientation dispersion (OD), microscopic apparent diffusion coefficient (microADC), cerebrospinal fluid volume fraction (V-CSF), and isotropic volume fraction (V-ISO) values significantly increased compared to NAWM. Wurtemberger et al. also found via a simple linear regression test that the average cellular density and ICVF have a moderate positive association (R^2^ = 0.32, *p* = 0.009), while MD and cellular density have a moderate negative association (R^2^ = 0.25, *p* = 0.03). A study by Genc et al. [52] aimed to detect the microstructural differences between left- and right-sided GBMs using various diffusion models. A widespread increase in MD for both groups was reported; however, the right-sided GBM group showed increased RD and AD on the ipsilateral side and increased ISO in contralateral white matter (WM). Contrarily, the left-sided GBM group showed increased mean kurtosis (MK) and axial kurtosis (AK) on the ipsilateral hemisphere, as well as increased orientation dispersion index (ODI) in the corpus callosum, though this effect was more widespread in the right-sided GBM group. Vikhrova et al. [50] used arterial spin labelling (ASL) metrics to compare tumour tissue metabolism with the genetic profiles of GBM. The GBM cohort was sub-grouped according to Ki-67 into two groups: one group for Ki-67 < 20% and another for Ki-67 > 20%. The mean CBF_max_ for the group with Ki-67 > 20% was 160.45 (SD = 46.01), and was 111.83 (SD = 66.26) for the GBM group with Ki-67 < 20% (*p* = 0.04). Zakharova et al. [57] aimed to determine the borders of malignant gliomas using diffusion kurtosis imaging (DKI) and pseudo-continuous ASL (pcASL) metrics. They computed a comprehensive panel of diffusional metrics, encompassing standard indices (e.g., FA, MD) alongside kurtosis-derived parameters (MK, AK, radial kurtosis [RK], kurtosis anisotropy [KA]) and compartmental models including axonal water fraction (AWF), axial extra-axonal space diffusivity (AxEAD), axial intra-axonal space diffusivity (AxIAD), radial extra-axonal space diffusivity (RadEAD), radial intra-axonal space diffusivity (RadIAD), tortuosity of extra-axonal space (TORT), as well as CBF for each of four defined ROIs per patient. ROIs were defined as follows: ROI1 was the CE tumour with the highest CBF, ROI2 was the perifocal infiltrative oedema, ROI3 was the NAWM around the tumour and along the surgical approach, and ROI4 was the contralateral NAWM. Zakharova et al. report that all of the samples obtained from ROI2 showed evidence of tumour cell infiltration, and that the Ki-67 LI and Bcl-2 antiapoptotic marker expression activity (EA) in samples from ROI2 were lower than in the samples from ROI1. It was found that ROI1 had the highest CBF values compared to the other ROIs (*p* < 0.001 for all), while there were significant differences detected between ROIs 2, 3, and 4. The comparison of ROI1 and ROI2 showed that AK, AxIAD, and RadIAD differed between the two ROIs (*p* < 0.001). Additionally, comparative analysis between ROI2 and ROI3 displayed differences in MD, FA, MK, RK, KA, AWF, RadEAD, and TORT (*p* < 0.001). Furthermore, FA, MK, RK, KA, AWF, and AxIAD between ROI3 and ROI4 also differed (*p* < 0.001). Zakharova et al. also performed correlational analysis by calculating the Spearman rank correlation coefficients which revealed the following significant (*p* < 0.05) correlations: in ROI1, Bcl2 EA correlated with AWF (rs = −0.504), AxIAD (rs = −0.385), FA (rs = −0.476), MD (rs = 0.421), RadIAD (rs = 0.444) and TORT (rs = −0.501); in ROI2, Ki-67 LI correlated with AK (rs = −0.434) and MK (rs = −0.381), while Bcl2 EA correlated with AWF (rs = −0.448), MK (rs = −0.522) and RK (rs = −0.497). They conclude that brain tissue that has been infiltrated by tumour cells possesses a more complex structure, owing to the presence of the tumour cells, gliosis, oedema, and neovasculature. Liesche-Starnecker et al. [64] used DTI and DSC metrics to investigate how tumour cellularity is reflected in MRI parameters. They report that correlation was apparent between CBV and cellularity (ρ = 0.129, *p* = 0.106). Meanwhile, MD and FA were negatively associated with cellularity (MD: ρ = −0.154, *p* = 0.50; FA: ρ = −0.095, *p* = 0.231). Kuroda et al. [44] analysed DWI and DSC-PWI data to increase the accuracy of radiologically differentiating between non-CE tumour (NET) and purely vasogenic oedema (pv-oedema). Kuroda et al. demonstrated that both cell density and the Ki-67 index were elevated in NET regions relative to pv-oedema (*p* < 0.05 and *p* < 0.01). Furthermore, when comparing NETs to the CE tumour, Ki-67 levels were higher in the latter (*p* < 0.05); however, cell density in the CE tumour and NET was comparable (*p* > 0.05). Similarly, there was no difference (*p* > 0.05) in the mean CBV ratios (values relative to the contralateral hemisphere) between the pv-oedema, NET, or CE tumour. It was found that the mean CBF ratio of pv-oedemas was lower (*p* < 0.01) than that of the NET. No notable difference was observed in CBF or mean transit time (MTT) between the CE tumour and NET. Additionally, the apparent diffusion coefficient (ADC) ratios did not differ significantly between CE tumour and NET, but the ADC ratio was higher (*p* < 0.05) in NET than in pv-oedema. Kuroda et al. were also able to perform stereotactic local comparisons between the imaging metrics and the cell density, Ki-67 LI, and micro-vessel area (MVA). The CBF ratio was found to correlate with both cell density (R = 0.400, *p* = 0.023) and Ki-67 LI (R = 0.374, *p* = 0.034). Similarly, the MTT ratio also correlated with the cell density (R = 0.409, *p* = 0.02) and Ki-67 LI (R = 0.322, *p* = 0.0003). There were no correlations between CBV ratios and the cell density or Ki-67 LI, or between the ADC ratio and the cell density or Ki-67 LI. These results were suggestive of DSC MRI’s potential to differentiate between NET and pv-oedema. Ying et al. [45] used multiple CEST-derived metrics (APTw, magnetisation transfer and nuclear Overhauser enhancement [MT&NOE], and CEST at 2 ppm) to assess the correlation between these CEST-derived metrics and both the cell density and proliferation potential in GBM. They utilised both typical (‘apparent’) methods and a quasi-steady-state (QUASS) algorithm for post-processing, resulting in two values for each derived metric. Ying et al. found that both Apparent_rAPT and QUASS_rAPT were positively correlated with both cell density (r = 0.588, *p* = 0.001, and r = 0.801, *p* < 0.001, respectively) and Ki-67 (r = 0.617, *p* < 0.001, and r = 0.776, *p* < 0.001, respectively). Meanwhile, both Apparent_ and QUASS_rCEST@2 ppm values showed no significant correlation with either cell density or Ki-67. Furthermore, Apparent_rMT&NOE values also showed no correlation with the cell density or Ki-67; however, QUASS_rMT&NOE was negatively correlated with both cell density and Ki-67 (r = −0.494, *p* = 0.009, and r = −0.527, *p* = 0.005, respectively). Ying et al. concluded that the QUASS algorithm improved the utility of APTw imaging for GBM. Rotkopf et al. [67] analysed DSC metrics to investigate the potential connection between wavelet-transformed magnetic resonance perfusion (wavelet-MRP) power spectrums and cell proliferation. Non-linear modelling of Ki-67 for both rCBV and wavelet-MRP values yielded insignificant correlations (*p* = 0.062 and *p* = 0.70, respectively). Rotkopf et al. also tested linear models for Ki-67 and either rCBV or wavelet-MRP, which demonstrated a similar lack of correlation (*p* = 0.992 and *p* = 0.899, respectively). From these results, tumour proliferation does not correlate with DSC-derived metrics. Sun et al. [56] used QSM and DWI and aimed to differentiate GBM subtypes. They found multiple significant positive correlations between the Ki-67 LI and QSM histogram features, including 90th percentile (P90), interquartile range (IQR), maximum value, median absolute deviation (MAD), root mean square (RMS), skewness, and variance (ρ ranged from 0.407 to 0.531, *p* < 0.01 for all). Sun et al. also reported negative correlations between the Ki-67 LI and both the 10th percentile (P10) of QSM (ρ = −0.452, *p* = 0.001) and P10 of ADC (ρ = −0.554, *p* < 0.001). They put forward QSM and ADC histogram features as potential imaging markers of the more intrinsic microstructure and physiology underlying GBM.

#### 3.2.2. Vasculature

The ten vasculature-focused studies are shown in Table 4.

Calvo-Imirizaldu et al. [42] examined pcASL with breath-hold challenge for CBF and cerebrovascular reactivity (CVR: measured as a function of signal change relative to the baseline CBF) quantification. Their analysis revealed compromised CVR within the tumour compared to contralateral grey matter (*p* = 0.0016). Additionally, a robust inverse relationship was observed within the tumour volume of interest (VOI) between baseline CBF and CVR values. (Spearman’s ρ = −0.71, *p* < 0.001). Calvo-Imirizaldu et al. also consolidated their previous findings that the CVR within the ipsilateral GM was lower than that of the contralateral GM; they deduce that it is likely a result of the impaired hemodynamic autoregulatory functions that are known to occur in GBM. Alvarez-Torres et al. [59] aimed to correlate DSC metrics with the MVA in GBM. Results showed the regions of the tumour with higher rCBV also possessed significantly larger micro vessel areas (rCBV_mean_: Spearman correlation = 0.38, *p* = 0.0008, rCBV_max_: Spearman correlation = 0.42, *p* < 0.0002). Additionally, Mann–Whitney tests revealed significant differences in rCBV_mean_ and rCBV_max_ between ROIs with and without micro vessels present (*p* = 0.0016 and *p* = 0.0005, respectively). Rotkopf et al. [67] analysed DSC-PWI data with the goal of elucidating the possible connection between the wavelet-MRP and the local vascularity in GBM. They report a significant association between the wavelet-MRP and the dichotomised CD31 expression in a mixed logistic model (*p* = 0.043), yet only an insignificant association between the CD31 expression and rCBV could be found (*p* = 0.297). Additionally, a linear association was found between the wavelet-MRP and the rCBV (Pearson’s correlation coefficient = 0.81). The results of a logistic regression model suggest that the wavelet-MRP is predictive of the local vessel density, while rCBV is not. Barajas et al. [73] evaluated whether quantitative DSC metrics are altered by the cellular and genomic expression patterns of GBM neoangiogenesis. Of the 57 pro-angiogenic genes of interest, 25 were differentially expressed between CE and NET regions (*p* < 0.05). Of these, 17 were found to be significantly upregulated in the CE regions, with an average fold change greater than 1.2. Barajas et al. also report that four of six angiogenesis pathways (VEGF-A: vascular endothelial growth factor A, PDGF: platelet-derived growth factor, HIF: hypoxia-inducible factor, FGF: fibroblast growth factor) were differentially expressed between CE and NET regions (*p* < 0.05, false discovery rate [FDR] < 0.05, B > 0). They also observed a strong correlation between VEGF-A-T1 expression and DSC-derived metrics (CBV, peak height [PH], percentage of signal intensity recovery [PSR]) (*p* < 0.05). Similarly, correlations between VEGF-A-T2 and both rCBV (r = 0.42, *p* = 0.03) and relative PSR (r = −0.42, *p* = 0.03) were found. These results indicate a direct correlation between the CBV and the molecular characteristics of GBM. Tykocinski et al. [75] observed that the relative tumour blood volume (rTBV) was not associated with lnVEGF (F-ratio = 2.71, *p* = 0.102). Dono et al. [48] investigated the role of radiographic features as predictors of 4q12 amplification in GBMs using DWI. They found that the diffusion characteristics differed significantly between 4q12-amplified and non-amplified GBMs (*p* = 0.00007; Benjamin–Hochberg FDR-adjusted *p* = 0.002). Dono et al. report that non-restricting and mixed patterns of diffusion comprised the majority of the 4q12-amplified GBMs (only 5% had a restricting pattern); conversely, non-amplified GBMs commonly had a restricting pattern (42.7%), rather than a non-restricting (29.2%) or mixed pattern (27.5%). They concluded that there are phenotypic imaging characteristics that can suggest clinically relevant molecular subtypes of GBM. Ulmer et al. [77] also utilised DSC to assess the effect of increased tumour perfusion on the ipsilateral tissue surrounding the tumour, compared to both within the tumour and the contralateral brain. Evidence indicated normal-appearing GM (NAGM) had higher rCBV and relative CBF (rCBF) values than NAWM (*p* < 0.001). Ulmer et al. also found a positive correlation for rCBV and rCBF between GM and WM regardless of hemisphere (affected or unaffected; *p* < 0.001). With respect to the NAGM, no difference was found between the affected and unaffected hemispheres for either rCBF or rCBV (*p* = 0.19 and *p* = 0.16, respectively). Ulmer et al. also found a robust correlation of the rCBF and rCBV values between the two hemispheres (rCBF: R = 0.98, *p* < 0.001; rCBV: R = 0.97, *p* < 0.001). They also reported that the rCBF and rCBV values in the tumour were significantly higher than the GM adjacent to the tumour (both *p* < 0.0001). There were also significant correlations for the two metrics between the tumour and the adjacent GM (rCBF: R = 0.69, *p* = 0.0009; rCBV: R = 0.85, *p* < 0.0001). Notably, these findings were independent of the size of the tumour and the distance between the tumour and NAGM. Ulmer et al. concluded that the increased perfusion within the tumour is not at the expense of that for the surrounding normal-appearing brain parenchyma.

Kuroda et al. [44] performed a multi-modal investigation using diffusion and DSC MRI to assess the characteristics of NET peripheral to the CE tumour as a means of increasing the diagnostic accuracy of MRI based on the histopathological differences between NET and pv-oedema. Stereotactic comparison revealed elevated NET microvessel area versus oedema (*p* < 0.05), with CE tumour demonstrating further MVA increases beyond NET values (*p* < 0.05). The mean CBV ratios between the CE tumour, NET, and oedema were comparable (*p* > 0.05); however, the mean CBF ratio for oedemas was notably lower than that of the NETs (*p* < 0.01). Kuroda et al. also found no significant difference in either the CBF or the MTT between the CE tumour and the NET; however, ADC ratios were found to be higher (*p* < 0.05) in the NET than in the oedema. Meanwhile, no difference (*p* > 0.05) was detected in ADC between the CE tumour and NET. Correlations between both the CBF ratio and the MTT ratio with the MVA were revealed (CBF: R = 0.443, *p* = 0.011; MTT: R = 0.430, *p* = 0.014); however, there were no correlations between the CBV ratio or ADC ratio with the MVA. Liesche-Starnecker et al. [64] used DTI and DSC-PWI metrics to improve our understanding of how neovascularisation is reflected in MRI parameters. Their evidence showed that CBV increased with the degree of neovascularisation, whereby analysis of variance (ANOVA) revealed a significant difference in CBV between areas of high and low neovascularisation (*p* = 0.003). Interestingly, Liesche-Starnecker et al. also found that FA values tended to be lower in areas of higher neovascularisation (*p* = 0.215).

Stumpo et al. [62] used functional (blood oxygen level-dependent: BOLD) MRI under different stimuli (hypercapnia, hypoxia, hyperoxia) to investigate the tumour tissue response patterns in new GBM patients. Their preliminary results were mixed for the seven recruited patients. Using maps that were binarised to either positive or negative %BOLD signal change, they assessed the mean %BOLD change in those voxels under the other two conditions. They observed no differences when using the binarised hypercapnic or hypoxic maps; however, the binarised hyperoxic maps showed a trend towards differential %BOLD signal change between positive and negative voxels during the other two stimuli. The mean %BOLD signal change during hypoxic stimuli in the hyperoxia negative voxels was less pronounced (−0.002 [SD = 0.009]) than in the positive voxels (−0.013 [SD = 0.006]). Additionally, the mean %BOLD signal change during the hypercapnia in the hyperoxia-negative voxels was of both opposite and greater magnitude (−0.055 [SD = 0.061]) than in the positive voxels (0.012 [SD = 0.054]). Interestingly, two patients were observed to experience only a negative %BOLD signal change during hypoxia, and only a positive signal change during hyperoxia. Meanwhile, the remaining five patients showed a negative %BOLD signal change during the hyperoxic stimulus in voxels near the tumour zones. Stumpo et al. concluded that these preliminary results reiterate the effects of GBM on the whole brain, thereby providing a basis for further investigations into the pathophysiology of tumour-related vascular and metabolic alterations.

#### 3.2.3. O6-Methylguanine-DNA Methyltransferase Promoter (MGMTp)

Table 5 presents the six studies investigating MGMTp methylation.

Ohba et al. [55] used mean and percentile APTw signals to evaluate whether MGMTp methylation status was associated with the APTw predictable diagnosis (of GBM vs. PCNSL). They report that there was no correlation between MGMTp methylation status and predictable diagnosis, nor a significant difference in the mean, first, and 100th percentile, or the width of 1–100 APTw signals between methylated and unmethylated MGMTp GBM. Chen et al. [43] used ADC histogram parameters from DWI to evaluate whether histogram analysis can predict the methylation status of the MGMTp. Performing histogram analysis of the whole tumour volume, they found that the ADC_min_ was lower (*p* = 0.005) in the MGMTp unmethylated subgroup compared to the methylated subgroup. No differences in any other histogram feature between the two groups were found. Chida et al. [47] aimed to determine the utility of MRI perfusion parameters for predicting the MGMTp methylation status in preoperative GBM patients. Regarding DSC metrics, tumours harbouring MGMTp methylation exhibited elevated (*p* < 0.005) rCBV compared to the unmethylated cohort. Similarly, mean rCBF values were significantly higher in tumours with MGMTp methylation than those without (*p* < 0.05); however, there was no significant difference in relative MTT (rMTT) between MGMTp methylated and unmethylated tumours. Chida et al. suggest that rCBV and rCBF are useful preoperative predictors of the MGMTp methylation status, which may make it possible to change the clinical management of GBM patients based on this knowledge. However, they do highlight the uncertainty of the combined prognostic value of these DSC metrics and MGMTp methylation status in terms of overall survival (OS) and progression-free survival (PFS) due to their limited sample size. Ladenhauf et al. [54] also used DSC MRI in combination with DWI to elucidate potential correlations between ADC values and MGMTp methylation status. They defined ROIs with corresponding contralateral ROIs as follows: (1) within the CE region of the tumour in the area of highest CBV (excluding necrotic components) (even ROIs are contralateral correspondent of the odd ROIs for normalisation of the respective values), (3) adjacent to ROI-1 beyond the CE region, (5) 2 cm from the CE region in NAWM, (7) NAWM distant from the CE region. Ladenhauf et al. observed higher ADC values in ROI-3 of the unmethylated MGMTp cohort compared to those with methylated MGMTp (*p* = 0.002). When normalising the ADC values, this effect was more pronounced (*p* = 0.0007). However, Ladenhauf et al. reported that no other ROI had a significant difference in ADC values (normalised or otherwise) between MGMTp methylated and unmethylated subgroups. Han et al. [69] utilised ADC and rCBF values to investigate the potential of these metrics for predicting the MGMTp methylation status of GBM patients. They used ADC values from ROIs placed in the solid tumour with the highest signal on DWI and the lowest ADC in combination with the rCBF values from ROIs similarly placed in the solid part of the tumour with the highest signal on the CBF map (normalised to contralateral). Their analysis revealed that the ADC values were lower in the unmethylated MGMTp group than in the methylated group (*p* < 0.001). Han et al. also found significantly higher rCBF values in the unmethylated group compared to those from the methylated group (*p* < 0.001). Vikhrova et al. [50] also examined the differences between methylated and unmethylated MGMTp GBM using DWI and ASL perfusion metrics. They reported significant differences only for the mean ADC_min_ between the two cohorts (*p* = 0.02).

#### 3.2.4. Epidermal Growth Factor Receptor (EGFR)

The results from the five studies investigating EGFR subtypes of GBM are shown in Table 6.

Young et al. [74] explored the potential of DWI features for predicting the EGFR amplification status of GBM patients. Quantitative DWI revealed EGFR-ADC associations, with amplified tumours displaying reduced ADC across all metrics (*p* < 0.01); the most significant correlations were found between EGFR status and the ADC values in the ROI (ADCROI: CE tumour with minimum ADC) (*p* = 0.0003) and the mean ADC values of the CE region (*p* = 0.0007). Gupta et al. [6] used DSC metrics to determine whether they could predict EGFR-defined subtypes of GBM. They reported that higher levels of EGFR amplification were associated with higher rCBVmedian (FDR-adjusted *p* = 0.03) and lower PSR (FDR-adjusted *p* = 0.052), while relative PH (rPH) did not differ significantly between EGFR-amplified and non-amplified GBM cohorts (*p* = 0.30). Gupta et al. also investigated the relationship between DSC metrics and the presence of the EGFR variant III (EGFRvIII) mutation. Analysis revealed an insignificant correlation between higher rPH and the presence of the EGFRvIII mutation (FDR-adjusted *p* = 0.10); however, no other relationship was elucidated between the remaining perfusion metrics and the EGFRvIII mutation (*p* > 0.19). Tykocinski et al. [75] derived the maximum rTBV using DSC to assess the accuracy of this metric for differentiating EGFRvIII-expressing subtypes of GBM in two cohorts, scanned by either a 1.5 T or 3 T scanner. Evidence indicated EGFRvIII-expressing GBMs possessed significantly higher rTBV in the CE region compared to those lacking the mutation in both cohorts (1.5 T: *p* = 0.001; 3 T: *p* = 0.000). Logistic regression analysis revealed that mean rTBV may act as a biomarker of EGFRvIII expression. In an integrated 1.5 T and 3 T analysis, Tykocinski et al. found that rTBV is an independent predictor of EGFRvIII expression (McFadden’s ρ^2^ = 0.23, *p* = 0.000), with an odds ratio of 2.70 (95% confidence interval, *p* = 0.000). However, Tykocinski et al. noted that despite the ability to predict the presence of the EGFRvIII mutation, EGFR wildtype cannot be predicted using rTBV. Vikhrova et al. [50] utilised DWI- and ASL-derived metrics (ADC and CBF) and compared this MRI data between genetic subtypes of GBM. They found no statistically significant correlation between any of the investigated MRI metrics and the EGFR status. Meanwhile, Bakas et al. [71] used DSC and DTI values to derive the ‘peritumoral heterogeneity index’ (PHI) (a measure of the separability between the summarised MRI measurements between two ROIs in a patient, where 0 indicates similar dynamics and one indicates different). They used the PHI as a means of detecting EGFRvIII mutations in GBM. Results showed that the difference in distributions of the PHI derived from DSC values between tumours with and without the EGFRvIII mutation was significant (*p* = 4.0033 × 10^−10^). However, the PHI derived from DTI metrics showed no ability to discriminate between tumours with and without the EGFRvIII mutation.

#### 3.2.5. Telomerase Reverse Transcriptase Promoter (TERTp) Mutations

Table 7 contains the summarised findings from the two studies investigating TERTp.

Using DWI, Kamimura et al. [49] investigated whether the TERTp mutation status of GBMs correlates with MRI features, with a focus on the ADC of the NET region. They found that TERTp-mutated (TERTp-mt) GBMs had a significantly lower ADC_min_ in the NET compared to the TERTp wildtype (TERTp-wt) cohort (*p* < 0.01); however, in the CE region, the ADC did not differ between TERTp-mt and TERTp-wt GBMs. They confirmed via histological examination that NET areas with low ADC experienced aggressive tumour invasion; however, only a trend towards a negative correlation could be observed between the ADC and tumour cell density. Additionally, sparse tumour cells were found in the NET with high ADC. Kamimura et al. suggest that TERTp-mt GBMs are more aggressive and have increased tumour cell invasion in the NET region. Chen et al. [43] also used DWI metrics, specifically, ADC histogram parameters, to evaluate whether they can predict the molecular characteristics of GBMs, including the TERTp mutation status. They computed the ADC histogram values for the whole tumour volume, reporting significant differences between the TERTp-mt and TERTp-wt groups in ADC_mean_, ADC_min_, and ADC_p10_ values (*p* = 0.003, 0.019, and 0.007, respectively). Additionally, results showed the entropy values were notably higher in the TERTp-mt group than those in the TERTp-wt group (*p* < 0.001).

#### 3.2.6. Immune Response

Five studies investigating the immune response in GBM are presented in Table 8.

Zhou et al. [58] examined DWI–tumour-associated macrophage (TAM) infiltration correlations, particularly M2-type TAMs (CD68-labelled TAMs, CD163-labelled M2-TAMs). The results showed CD163+ macrophage infiltration was associated with the ADC_mean_ of the CE region (r = 0.208, *p* = 0.014); however, ADC_min_ was not correlated with TAM infiltration. Their results support the notion that water diffusion is more restricted as tumour cells continue to proliferate. Zhang et al. [46] aimed to assess the prognostic significance of programmed death-ligand 1 (PD-L1) expression in GBM and explored whether MRI-derived Visually AcceSSible Rembrandt Images (VASARI) features could predict PD-L1 status preoperatively. PD-L1 expression was quantified using IHC and reported as a tumour proportion score across eight ROIs. Among 124 patients (71 low PD-L1, 53 high PD-L1), they report that the VASARI feature F5 (proportion of enhancing tumour) was higher (*p* = 0.003) in the low PD-L1 expression group compared to the high expression group, while all other VASARI features showed no differences (*p* > 0.05). Diffusion characteristics, categorised as restricted, facilitated, or mixed, did not differ between PD-L1 expression cohorts (*p* = 0.722). These findings suggest that although PD-L1 expression negatively correlates with OS, it does not appear to influence diffusion properties, and only limited morphological features, such as enhancement proportion, may reflect PD-L1 status.

Rohrich et al. [66] combined DWI, DSC PWI, and fibroblast activation protein (FAP)-specific PET imaging to correlate MR findings with the FAP-specific signalling in GBM. Moderate positive FAP signalling–rCBV associations emerged within NET territories (r = 0.229), with a smaller effect noticed in the CE region (r = 0.09). No correlation was found when analysing FAP-specific signalling and ADC values for either the NET (r = 0.038) or the CE region (r = 0.017). Their results support the role of FAP-specific PET as a complementary modality to MR for GBM imaging. Nakamura et al. [60] used MRS with a short echo time (TE = 35 ms) to assess how metabolite concentration ratios (Glu: glutamate; NAA; Lac: lactate; all relative to Cr) differ between GBM patients who experience different types of seizures, and have different expression levels of CD44 (expressed as a periphery-to-core [P/C] ratio). They positioned MRS voxels beyond the CE region, in areas of noticeable 11C-MET-PET uptake, and classified patients into subgroups according to the characteristics of seizure onset (type-A: no seizures before or after treatment; type-B: seizure before but not after treatment; type-C: seizures after treatment, regardless of whether seizures occurred prior to treatment). Nakamura et al. reported that GBMs with a highly invasive radiological phenotype had a higher mean P/C ratio of CD44 expression than the low invasive phenotype (*p* = 0.027); however, CD44 expression differed insignificantly between subgroups with (type-B + type-C) or without seizures (type-A) (*p* = 0.10), or between types A, B, and C. They also observed that the group experiencing seizures (type-B + type-C) had significantly higher Glu/Cr and NAA/Cr than those that did not (type-A) (*p* = 0.011 and *p* = 0.007, respectively), while Lac/Cr did not differ significantly. When comparing each type individually, Nakamura et al. found that type-C had significantly higher Glu/Cr, NAA/Cr, and Lac/Cr than type-A (*p* = 0.002, *p* = 0.037, and *p* = 0.0003, respectively); meanwhile, only NAA/Cr differed significantly when comparing type-B and type-A (*p* = 0.047). Additionally, higher Lac/Cr was the only significant difference between type-B and type-C (*p* = 0.0002). Observations revealed that all of the GBMs in the type-B cohort were low-invasive phenotype, while type-C consisted only of highly invasive phenotype GBMs. They extended upon this analysis and found that type-C had higher (*p* < 0.05) expression of CD44 compared to type-B, suggesting that the high expression of CD44 on GSCs, which inadvertently persists after treatment in highly invasive phenotypes, may be responsible for both tumour recurrence and seizures that occur post-treatment.

Nazem et al. [61] used a combination of DSC, QSM, and R2* metrics to quantify and elucidate whether iron may be a biomarker of TAMs in GBM. They reported a significant positive correlation between QSM-based mean susceptibility in the CE region and the ferritin light chain (l-ferritin) positivity percent from the representative tissue section (r = 0.56, *p* = 0.7). Nazem et al. also observed significant correlations between the mean susceptibility of the CE region and both CD68 positivity (ρ = 0.52, *p* = 0.034) and CD86 (ρ = 0.7, *p* = 0.001), but notably not for CD206 (ρ = 0.09, *p* = 0.7). Further analysis of the combined mean susceptibility from the CE and necrotic regions revealed correlations with l-ferritin (r = 0.72, *p* = 0.001) and CD86 positivity (r = 0.63, *p* = 0.005). Meanwhile, only a trend was observed between combined mean susceptibility and CD68 positivity (r = 0.46, *p* = 0.06). Their results are supportive of the roles of M1-type TAMs in storing intracellular iron and M2-type TAMs in releasing iron into the tumour microenvironment (TME).

#### 3.2.7. Tumour Microenvironment

Table 9 includes results from the two studies investigating the TME.

Stadlbauer et al. (2018) [70] used a physiological MRI protocol comprising quantitative BOLD and vascular architecture mapping (VAM) to investigate hypoxic and vascular niches within the TME. They also extended this to correlate these niches with the primary metabolic pathway for energy production as a means of distinguishing between subgroups of GBM. Using this approach, Stadlbauer et al. classified six different TME profiles according to the MRI-derived oxygen extraction fraction (OEF) and cerebral metabolic rate of oxygen (CMRO_2_; measured via mitochondrial partial pressure of oxygen [mitoPO_2_]) and degree of neovascularisation: necrosis, hypoxia with/without neovascularisation, oxidative phosphorylation (OxPhos), and glycolysis with/without neovascularisation. They found that the volumetric composition in terms of the TME allowed two GBM phenotypes to be differentiated, one maintained primarily via glycolysis with mostly functional neovascularisation, and another dominated by necrosis and hypoxia with defective neovascularisation. Between these two groups, the volumes of all defined TMEs, besides OxPhos with neovascularisation, differed significantly (*p* < 0.005). Furthermore, Stadlbauer et al. reported a common spatial structure of the TMEs—centralised necrosis surrounded by hypoxic tumour, with either defective or functional neovascularisation, and a dominant TME of either OxPhos or glycolysis. They conclude that their method allows the non-invasive detection of tumour-supportive niches, which could potentiate improved clinical responses for patients. Stadlbauer et al. (2021) [65] extended upon their previous work utilising physiologic MRI metrics to determine if TME mapping can provide meaningful insights into the pathophysiologic differences between CE brain tumours of various origins. Results showed that in the vital tumour, defined as the sum of the aerobic glycolysis and oxidative phosphorylation TMEs, approximately two-thirds used aerobic glycolysis for energy production. Specifically aerobic glycolysis comprised 37% ± 22% of the GBM TME, while OxPhos contributed 17% ± 6%, vital tumour contributed 54% ± 24%, necrosis contributed 22% ± 11%, hypoxia with neovascularisation contributed 15% ± 10%, hypoxia without neovascularisation contributed 9% ± 7%, and the total contribution of hypoxia (with and without neovascularisation) was 24% ± 16%.

#### 3.2.8. Statistical Heterogeneity

Across included studies, statistical and methodological heterogeneity was substantial, precluding quantitative pooling. Effect metrics varied: many studies reported non-comparable correlations (Pearson vs. Spearman; unadjusted vs. adjusted) and used different target endpoints. Furthermore, ROI definitions and methods of normalisation differed—for example, hotspot vs. whole-lesion ROIs, 2D vs. 3D ROIs, and normalisation to different NAWM regions (either direct contralateral correspondent or a representative contralateral region)—all of which have an effect on the measured values and subsequent diagnostic performance. Additionally, the acquisition parameters, field strengths, and processing pipelines were inconsistent across modalities and between studies. Collectively, the differences in this body of evidence indicate that directionality and qualitative consistency of associations are more reliable than absolute effect magnitudes.

## 4. Discussion

### 4.1. Summary of Evidence

This scoping review found 37 articles that contribute to answering the question “can advanced MRI help us understand the biology of GBM”, by summarising what is known about the biological implications of findings generated by advanced MRI techniques used to preoperatively image GBM patients. The biological domains covered in this scoping review include brain microstructure, vasculature, MGMTp, EGFR, TERTp mutations, the immune response, and the TME. Across the 37 studies, advanced MRI metrics showed associations with proliferation (Ki-67), angiogenesis (e.g., VEGF, MVA), metabolic proxies (Cho-based ratios), selected genomic alterations (MGMTp, EGFR, TERTp), and immune markers (e.g., CD68, CD86, CD163); effect sizes and consistency varied by modality and method.

In response to RQ2, our synthesis suggests that while QSM offers unique links to immune biomarkers, both MRS and APTw imaging demonstrate the most reliable correlations with metabolism and cellular proliferation. Furthermore, DWI and PWI, while widely used, show variable specificity, likely a reflection of methodological heterogeneity, including differences in acquisition protocols, ROI placement, and post-processing algorithms. This highlights the need for harmonised protocols and the benefit of multimodal integration where single-modality approaches seem insufficient for biomarker development. Field strength is another source of methodological heterogeneity that contributes to variability in quantitative metrics throughout the included articles. Across included studies, most were 1.5 T and 3 T, with a small minority at 7 T. The influence of these effects is largely practical rather than mechanistic, and as such, we therefore synthesise associations narratively rather than pooling across field strengths for each modality and biological domain. QSM and physiologic MRI show notable potential for insights, yet are underrepresented in the current literature. Meanwhile, emerging techniques such as MR fingerprinting, MR angiography, and MR thermometry were entirely absent from the included studies, suggesting opportunities for future exploration using these novel methods. This review revealed that the TERTp status, immune response, and TME remain insufficiently characterised by advanced MRI methods. Few studies integrate imaging with the mutation status of the TERTp, immune markers (PD-L1), or GSC biology, indicating potential gaps, potentially arising from lower clinical relevance.

The typical limitations of the aforementioned studies include small sample sizes, single-centre designs, heterogeneous imaging protocols, and a lack of standardisation in post-processing, which each contribute to reducing the generalisability of any findings. Many studies also fail to account for confounding factors such as tumour size, necrosis, and treatment variability. Notably, the 2021 WHO classification was adopted in the majority of eligible studies, minimising the risks of inferring the IDH mutation status.

#### 4.1.1. Brain Microstructure: Proliferation and Invasion

The microstructure of the brain is known to be impacted by GBM-related cell proliferation and migration. This proliferative and invasive nature of GBM is one of the most investigated and well-documented features of the malignancy, yet it persists as a significant challenge. Research into the non-invasive profiling of the microstructure using MRI has yielded mixed results and hence, is ongoing. Across the 15 eligible studies, advanced MRI techniques based on the properties of diffusion and perfusion have been the most commonly utilised for potential utility in improving the radiologic evaluation of the tumour; however, APTw imaging and MRS have also been tested. They have significantly deepened our understanding of GBM biology, particularly with respect to tumour proliferation, invasion, and metabolic heterogeneity.

APTw imaging has shown some of the strongest promise in non-invasively characterising GBM biology, particularly proliferation. Multiple studies demonstrate correlations between APTw signals and Ki-67, supporting its role as a marker of cellular activity in the tumour [45,53,55]. Yet the consistency of these findings is lacking; mean values seem to better capture proliferative potential than percentile-based analyses, which often add little discriminatory power. While this modality excels in mapping metabolically active GBM regions, differences in post-processing methods highlight the need for technical standardisation. However, overall, APTw showed repeated associations with proliferation indices, but technical harmonisation is essential before it can be considered for translation into the clinic.

Studies integrating MRS with CEST [63] found correlations with proliferation markers using combined probability maps that outperform either technique alone; however, patient-level performance was inconsistent, suggesting MRS is most informative as part of a multimodal protocol. Robust associations between Cho/Cr values and histological proliferation markers reinforce its utility. However, patient-level correlations remain inconsistent, while voxel-wise metabolic mapping is powerful, the inter-patient variability limits its standalone predictive value. As such, MRS appears most effective when integrated into a multimodal approach, where it acts as a metabolic complement.

Diffusion-based techniques highlight the microstructural disruption, characteristic of GBM, through varied results [44,50,51,52,56,57,64,72]. Reduced anisotropy and increased diffusivity in tumour regions were consistently reported, aligning with expectations of tissue disorganisation. Furthermore, mean diffusivity shows constant negative associations with cellular density, while kurtosis measures produce variable results dependent on the ROI and/or tumour laterality. Hemispheric differences were shown to further complicate the interpretation, suggesting that diffusion profiles may depend on network-level factors beyond tumour infiltration alone. Thus, while DWI was sensitive to GBM-related microstructural change, biological specificity was variable across acquisitions and methods of analysis.

PWI yielded the most inconsistent findings in this literature [44,50,57,64,67,72,76]. Some studies demonstrate associations between elevated CBF and proliferation indices, while others report no meaningful correlation. Theories behind these discrepancies are that perfusion primarily reflects vascular remodelling, which does not uniformly track with tumour cellularity. Notably, while rCBV is often considered a gold-standard perfusion marker, multiple reports highlight its weak or even absent correlation with histological features. Nevertheless, PWI remains clinically useful for mapping vascular heterogeneity.

QSM facilitated the derivation of histogram features that show consistent and robust correlations with proliferation markers, suggesting that iron deposition and microvascular alterations may be better proxies of GBM biology than haemodynamics alone [56]. These QSM features exhibited reproducible associations in limited cohorts and, once validated across larger cohorts to establish their reliability and optimise their integration into multimodal imaging strategies, may be considered a candidate for future clinical integration.

Taken together, these advanced MRI techniques provide partial, complementary windows into GBM biology. CEST and MRS appear most reliable for assessing metabolic activity and proliferation, DWI excels in detecting microstructural disruption but lacks biological specificity, and perfusion remains inconsistent in its correlation with tumour aggressiveness. QSM stands as a potentially reproducible approach for linking imaging to histology, yet it requires considerably more research. Greater utility arises from multimodal integration; hence, future research would benefit from moving toward standardised protocols and integrated models to translate these radiologic biomarkers into clinical practice.

#### 4.1.2. Vasculature

GBM typically exhibits dysfunctional vasculature [78], which plays a central role in tumour growth, invasion, and treatment resistance. Driven by hypoxia and overexpression of angiogenic factors such as VEGF, GBM induces a dense network of structurally irregular vessels that are leaky and poorly organised, resulting in heterogeneous blood flow and impaired oxygen delivery. These vascular abnormalities create regions of hypoxia that promote tumour aggressiveness and therapy resistance. Clinically, vascular features are key imaging biomarkers for diagnosis and monitoring, and they serve as therapeutic targets for anti-angiogenic agents like bevacizumab, although benefits are often transient. Moreover, vascular density and perfusion heterogeneity correlate strongly with prognosis, making vasculature a critical determinant of GBM biology and patient outcomes.

PWI consistently emerges as a key tool for assessing tumour vascularity and angiogenesis, with several studies demonstrating its correlation with histopathological markers [42,44,59,64,67,73,75,77]. Numerous studies revealed that regions of high rCBV are associated with increased MVA and CD31 expression, reinforcing its potential as a surrogate for angiogenesis. Recent developments have also nominated wavelet MRP metrics as an improved predictor of local vessel density, compared to rCBV, suggesting that advanced signal processing techniques could enhance diagnostic accuracy, since conventional PWI metrics may not fully capture the complexity of GBM vasculature. Alternatively, this divergence may arise from differences in the spatial resolution of the techniques or even the heterogeneity of GBM itself. Areas of high rCBV reflect active angiogenesis; however, PWI alone seems unable to distinguish between functional and non-functional vessels.

Calvo-Imirizaldu et al. [42] introduced a novel approach by assessing CVR as opposed to the CBF or CBV in GBM. By measuring vascular responsiveness rather than static blood volume, their report supports the understanding that autoregulatory haemodynamic functions are impaired in GBM, as well as beyond its detectable infiltration. Most notably, they found an inverse correlation between baseline CBF and CVR, which they state is likely due to the disorganisation of the immature neoangiogenic vessels. Interestingly, one early study found that the increased perfusion within GBM does not compromise that of the surrounding normal-appearing brain tissue, which is suggestive of a compartmentalised vascular response.

DWI has also shown utility in interpreting the vascular environment in patients with GBM [44,48,64]. Qualitative diffusion patterns were demonstrated to differ significantly between 4q12-amplified and non-amplified GBMs—4q12 being a chromosomal region that harbours receptor tyrosine kinase genes, which are frequently amplified in GBM and associated with aggressive tumour behaviour. The combination of perfusion and diffusion characteristics appears more promising. By integrating DTI metrics insights regarding how the neovasculature affects the integrity of the WM become available. Observations by Liesche-Starnecker et al. [64] of reduced FA in highly vascularised regions suggest that neoangiogenesis coincide with microstructural disruption, likely due to GBM infiltration—reiterating the notion that vascular proliferation in GBM is not merely a passive process but actively remodels the surrounding brain.

BOLD MRI was employed alongside the application of various physiological stimuli to investigate GBM-related vascular alterations [62]. This approach revealed heterogeneous BOLD responses, and this variability across patients and stimuli aligns with the widespread impact GBM has on brain physiology; however, the results are likely complicated by differences in tumour location, vascular architecture, and metabolic demand. Despite this, these whole-brain effects reinforce the idea that GBM is not confined to its enhancing margins but exerts systemic influence on cerebral function.

Overall, these studies illustrate both the promise and limitations of perfusion and diffusion metrics for gaining insights into the vascular and microstructural features, accompanied by the advisement that their interpretation must be considered in the appropriate biological context.

#### 4.1.3. O6-Methylguanine-DNA Methyltransferase Promoter (MGMTp)

The MGMTp methylation status is routinely used for prognosing the response of GBM patients to the standard chemotherapy treatment, TMZ [24]. The methylation of the MGMTp limits the expression of the MGMT protein, which is responsible for DNA repair [23]. Hence, GBMs with MGMTp methylation are more sensitive to the alkylating agent TMZ, while those with unmethylated MGMTp are resistant. While this has seemingly clear clinical relevance, the current methods used to determine the methylation status yield late results and are invasive. Thus, non-invasive means of determining the MGMTp methylation status using advanced MRI techniques would stand to overcome both of these current limitations.

Among six eligible studies, DWI metrics showed the most consistent findings, differing significantly between methylated and unmethylated MGMTp subgroups of GBM. The minimum ADC of unmethylated MGMTp GBMs was significantly lower than that of those with methylated MGMTp [43,50,69], likely reflective of an increased cellularity. However, a single study [54] reported that the absolute and normalised ADC values in the peritumoural region (beyond/adjacent to the CE tumour) were significantly higher in MGMTp unmethylated GBM than those that were methylated. ADC maps of GBM are typically interpreted as reflections of the cellularity, as they reflect the motion of water molecules in tissue. Hence, a lower ADC equates to the restriction of diffusion in the tissue, which implies a higher cellularity (the opposite is also true). This relationship has been supported by histopathological correlations in several studies, where regions of low ADC corresponded to densely packed tumour cells. These findings align with the broader understanding that lower ADC values typically correspond to increased tumour cellularity, which may in turn reflect the more aggressive phenotype often associated with MGMTp unmethylation. Based on this evidence, it stands to reason that DWI-derived ADC metrics could be implemented clinically, perhaps after further validation between vendors and field strengths, since DWI is already a component of many clinical neurological workups.

One possible explanation for the variability of these findings could be the result of differing methodologies in relation to DWI acquisition, ROI placement, or field strength, or perhaps the complexity of the inherent heterogeneity of GBM, all of which render comparison difficult. To help elucidate the true relationship between the MGMTp methylation status and ADC values, further multi-site studies or a meta-analysis are warranted.

PWI has also been investigated for its potential utility in predicting the methylation status of the MGMTp in GBM. Current reports present disparate findings; Chida et al. [47] report that DSC-derived rCBV and rCBF values of the CE region (normalised to contralateral) were significantly higher in MGMTp-methylated GBMs than in the unmethylated cohort; however, rMTT values did not differ significantly between groups. Meanwhile, pcASL-derived rCBF_max_ values from the solid portion of GBMs (CE + peritumoural oedema) are significantly higher in tumours with an unmethylated MGMTp [69].

Since PWI measures flow and volume of blood relative to the contralateral ‘unaffected’ hemisphere (rCBF and rCBV), it is thought to reflect the degree of neoangiogenesis induced by the tumour. It has been shown that MGMTp methylation is positively correlated with the expression of VEGF, suggesting that GBMs with a methylated MGMTp may have a propensity towards more structured neovasculature, as indicated by the higher rCBV and rCBF derived from DSC.

However, the ASL-derived rCBF_max_ was reported to have the opposite relationship with the methylation status (lower rCBF_max_ in the unmethylated subgroup). This could again be a result of the unstandardised acquisition methods, ROI placement, or the derivation of metrics (one study looks at the maximum value of rCBF, while the other looks at the value in a specified location). This discrepancy may also reflect the influence of confounding factors such as tumour size, necrosis, and vascular heterogeneity.

CEST imaging was implemented in a single study for the determination of the MGMTp methylation status [55]. Ohba et al., 2023 [55] found no significant difference in percentile values of the APTw signal from the whole tumour (as defined by the CE) between methylated and unmethylated MGMTp subgroups. This suggests that APTw imaging may not be suited to detecting the underlying metabolic signatures that differentiate between MGMTp methylated and unmethylated GBMs. However, the utility of APTw imaging in other facets of neuro-oncologic imaging warrants further investigation, via larger studies, into its potential for GBM subtyping.

#### 4.1.4. Epidermal Growth Factor Receptor (EGFR)

EGFR amplification is present in more than 50% of GBMs [25]. EGFR is a receptor tyrosine kinase with downstream pathways responsible for DNA synthesis and cellular proliferation; hence, the amplification of this receptor promotes tumour invasion and drug resistance. EGFR mutations can also cause structural variants, such as the EGFRvIII variant that is found only in tumours, including GBM. EGFRvIII is found in about 50% of EGFR-amplified GBMs and confers an even more aggressive phenotype. Methods of detection for molecular alterations of the EGFR gene currently rely on tissue samples obtained from patients during their surgery to remove the tumour or obtain a biopsy, the results of which can often arrive too late to inform treatment. MRI-based methods for predicting the molecular alterations present in the EGFR gene have been investigated, and numerous correlations between advanced MRI metrics and EGFR status have been explored.

A total of five eligible studies applied advanced MRI techniques in a cohort of GBM patients and investigated EGFR-related molecular alterations. These studies utilised either PWI, DWI, or a combination of the two to do so; these two modalities are logical choices since EGFR is linked to the expression of VEGF. The use of DWI for predicting between the amplified and non-amplified EGFR GBMs has yielded mixed reports [71,74]. An early investigation found that EGFR amplified GBMs had decreased (all *p* < 0.01) ADC-derived metrics (mean, max, min, ROI) compared to the non-amplified cohort [74]. They also observed correlations between the EGFR status and the ADC_mean_ and ADC_ROI_ metrics specifically. However, a more recent report, looking only at patients over 50 years of age, failed to detect any significant difference between EGFR-amplified and EGFR-non-amplified patients.

These opposing findings are likely a reflection of different methodologies and the cohort of interest; while both were investigating GBM, the latter explored the EGFR amplification status only in the older subgroup. The inconclusive nature of these findings mandates further investigation using these two modalities.

PWI has also been found to have mixed findings on the impact of EGFR amplification and the EGFRvIII mutation. Gupta et al. [6] and Tykocinski et al. [75] suggest that EGFR-driven biology influences vascular characteristics, but they differ in which metrics capture these effects. Gupta et al. [6] found that EGFR amplification is associated with higher rCBV and lower PSR, indicating increased vascularity and reduced vessel stability, yet rPH and EGFRvIII showed no strong associations. In contrast, Tykocinski et al. [75] demonstrated that rTBV—a metric reflecting extreme perfusion values—robustly predicts EGFRvIII expression, suggesting that this variant may drive focal regions of intense angiogenesis rather than global perfusion changes. This discrepancy could reflect differences in metric sensitivity: rCBV and PSR capture average perfusion and leakage dynamics, whereas rTBV highlights vascular extremes, which may be more relevant for understanding EGFRvIII.

Vikhrova et al. [50] reported no correlation between EGFR status and diffusion or ASL-derived metrics, reinforcing that EGFR-related changes are not primarily structural or baseline perfusion phenomena but rather dynamic vascular alterations. Finally, Bakas et al. [71] introduced PHI, showing that perfusion heterogeneity strongly discriminates EGFRvIII-positive tumours, whereas diffusion-based PHI does not, aligning with Tykocinski’s [75] emphasis on vascular extremes and heterogeneity. These results indicate that EGFR amplification broadly increases vascularity, while EGFRvIII may drive more heterogeneous and extreme perfusion patterns. Clinically, this implies that advanced DSC-derived metrics (rTBV, PHI) could serve as non-invasive biomarkers for EGFRvIII, whereas standard perfusion measures like rCBV may better reflect amplification status. However, the variability across studies indicates the need for harmonised imaging protocols and multi-parametric approaches to reliably capture EGFR-driven vascular phenotypes.

#### 4.1.5. Telomerase Reverse Transcriptase Promoter (TERTp) Mutations

Mutations in the TERTp are among the most common molecular alterations in GBM [26,27], driving increased telomerase activity and promoting tumourigenesis—contributing to immortalisation and GBM’s aggressiveness. Given its contribution, the non-invasive identification of these mutations could aid in the diagnostic workup of new GBM patients, likely in the complement of current molecular methods of determination.

DWI was the prime modality used when searching for MRI-based biomarkers of the TERTp status. Kamimura et al. [49] reported that TERTp mutant GBMs showed reduced ADC_min_ values in the NET region compared to those without the mutation (*p* < 0.05); however, no notable difference was observed in the CE region between the two groups [49]. Histological analysis confirmed that the NET regions with low ADC_min_ corresponded to areas of aggressive invasion, while the NET regions with higher ADC_min_ contained sparse tumour cells. Their findings suggest that TERTp mutations are associated with the infiltrative behaviour in the more peripheral, leading edge, of the tumour, which is readily apparent using conventional MRI. This work was expanded upon by Chen et al. [43] who found significantly lower ADC_mean_, ADC_mean_, and ADC_10th percentile_ values in the TERTp mutant GBM group. These results support the notion that TERTp mutations confer a more invasive phenotype that is detectable using DWI metrics.

Overall, DWI appears capable of detecting the changes caused by TERTp mutations and is sensitive to its invasive biological signature. The consistent findings between the two studies suggest that this advanced MRI modality can act as a useful, non-invasive predictor of TERTp status. However, future studies should also replicate and validate this in larger cohort studies using a more standardised approach to analysis.

#### 4.1.6. Immune Response

The immune response in GBM, once thought negligible due to the CNS being considered immune-privileged [79,80], is now recognised as a critical determinant of tumour progression and therapeutic resistance [80,81]. However, evaluating the immune microenvironment remains challenging, and imaging biomarkers offer a promising non-invasive approach.

Across studies, correlations between imaging metrics and immune-related markers reveal both consistent patterns and notable variability. Zhou et al. [58] demonstrated that M2-type TAM infiltration (CD163+) is significantly associated with ADC_mean_ in the CE region, suggesting that increased macrophage presence coincides with restricted water diffusion, likely reflecting dense cellular proliferation. In contrast, Nazem et al. [61] focussed on iron metabolism as an immune-related feature, reporting strong correlations between QSM-derived susceptibility and markers of M1-type TAMs (CD86) and ferritin, but not M2-type TAMs (CD206), supporting the hypothesis that M1 macrophages store intracellular iron while M2 macrophages facilitate iron release into the tumour microenvironment. These findings highlight the potential of QSM as a biomarker for macrophage phenotype and iron dynamics in GBM. Rohrich et al. [66] extended this concept by integrating DSC and FAP-specific PET imaging, revealing moderate correlations between FAP signalling and rCBV, particularly in NET regions, while diffusion metrics showed no association. This suggests that FAP expression, linked to stromal activation and immune modulation, may drive vascular remodelling rather than microstructural changes, positioning FAP-PET as a complementary modality to PWI. Meanwhile, Zhang et al. [46] explored PD-L1 expression, a key immune checkpoint molecule, finding that high PD-L1 levels negatively correlate with OS but show no association with diffusion characteristics, and only limited correlation with morphological features such as enhancement proportion. Finally, Nakamura et al. [60] investigated CD44 expression and seizure phenotypes, observing that highly invasive GBMs exhibit elevated CD44 and altered metabolite profiles, particularly in patients with post-treatment seizures, implicating GSC persistence and immune-related signalling in recurrence and symptomatology. Collectively, these studies underscore that immune-related markers in GBM manifest through diverse imaging signatures—perfusion heterogeneity for FAP, susceptibility for iron-rich TAMs, and metabolic shifts for CD44—while diffusion appears less sensitive to immune modulation. Clinically, this suggests that multi-modal imaging, integrating PWI, SWI, and PET, may provide a more comprehensive assessment of the immune microenvironment, informing prognosis and guiding immunotherapeutic strategies.

#### 4.1.7. Tumour Microenvironment (TME)

The TME in GBM comprises a complex interplay of cellular, vascular, and metabolic components that collectively influence tumour growth, invasion, and therapeutic response. Beyond tumour cells, the TME includes hypoxic niches, necrotic regions, and areas of variable vascular functionality, all of which shape metabolic pathways and treatment resistance. Non-invasive imaging approaches have recently enabled the characterisation of these microenvironments in vivo, offering insights into GBM heterogeneity and potential therapeutic targets.

Stadlbauer et al.’s work [65,70] demonstrates that physiologic MRI can non-invasively map metabolic and vascular niches, revealing distinct GBM phenotypes driven by either glycolysis with functional neovascularisation or by necrosis and hypoxia with defective vasculature. These findings have significant implications: first, they highlight that GBM metabolism is not uniform—while aerobic glycolysis predominates, oxidative phosphorylation persists in some regions, suggesting that metabolic targeting strategies must account for this heterogeneity. Secondly, the spatial organisation of TMEs, with central necrosis surrounded by hypoxic zones and variable vascular integrity, underscores the challenge of drug delivery and the potential need for therapies that normalise vasculature or exploit hypoxic vulnerabilities. Furthermore, the ability to classify GBM phenotypes based on metabolic and vascular profiles could enable personalised treatment approaches, such as selecting patients for anti-angiogenic therapy, hypoxia-targeted drugs, or metabolic inhibitors. Collectively, this work highlights physiologic MRI’s potential as a powerful tool for precision oncology in GBM, bridging the gap between imaging and molecular pathology.

### 4.2. Clinical Relevance

Despite the technical promise of many advanced MRI techniques, few have achieved clinical integration—a result of practical considerations such as scan duration, cost, ease of acquisition, required expertise, extent of post-processing required, and interpretability. These considerations must guide future research, since the potential benefits for both patients and the clinicians treating them include the non-invasive identification of clinically actionable insights, particularly as new therapies are developed and tested in clinical trials, which will also require a means of screening and stratifying patients for response to the novel therapeutic. Advanced imaging research and developments need to accompany that of emerging therapeutics to ensure that a personalised, precision medicine approach is available. To work towards this, these MR techniques require optimisation for routine workflows.

Taken together, the techniques closest to routine clinical adoption for preoperative GBM are DTI and PWI, particularly DSC, both of which are already used in many centres to inform surgical planning and to aid diagnosis. DTI provides reproducible microstructural correlates that assist with tumour characterisation and cellularity assessment, and is recognised as a complement to conventional anatomical MRI for GBM care [17]. MRS occupies an intermediate position, clinically available but limited by technical complexity and a lack of standardised interpretation criteria that restrict routine adoption. By contrast, APTw and QSM show strong promise for biology-specific readouts (protein content and iron/calcification, respectively), yet they currently remain in the research domain because inter-site standardisation of acquisition and post-processing is still evolving. As such, DTI and DSC-PWI seem to provide the most reproducible, clinically translatable correlates today, whereas CEST/APTw and QSM remain research-leaning pending standardisation and validation.

### 4.3. Limitations

This review has several limitations that warrant consideration. First, the assumption that pre-2021 reports of de novo or primary GBM equate to IDHwt status may introduce classification bias, despite clinical evidence supporting this equivalence—notably, only seven eligible articles required an inference of the IDH mutation status. Second, most included studies were single-centre with small sample sizes, limiting generalisability and statistical power. Third, heterogeneity in imaging protocols, field strengths, and post-processing methods precluded meta-analysis and complicates direct comparisons across studies. Additionally, retrospective designs often lacked precise correlation between imaging regions of interest and histopathological sampling, which may affect the accuracy of reported associations. The review was also restricted to articles accessible via institutional subscriptions and published in English, potentially excluding relevant evidence and introducing language and geographic bias. Grey literature was excluded, which may omit emerging or preliminary data on novel imaging approaches. Furthermore, data extraction was primarily performed by a single reviewer, introducing a risk of bias and extraction error, which is partially mitigated by the partial validation of 30% of the eligible records by a secondary reviewer. Studies employing ML, AI, or radiomics were excluded from the scope of this review to preserve a biological grounding in eligible articles, which may limit insights into emerging analytical approaches, such as those that utilise interpretable models. We acknowledge that this removes a major contemporary body of evidence; however, it would benefit from a narrow, focused synthesis of ML, AI, and radiomics approaches to investigating GBM; research that currently exists already covers a large portion of the field [82,83,84,85,86,87,88,89,90]. Finally, confounding factors such as tumour size, necrosis, and treatment variability were rarely accounted for in the included studies. Future research should prioritise multi-centre collaborations, harmonised acquisition and analysis protocols, and prospective designs to validate and standardise imaging biomarkers for clinical translation.

## 5. Conclusions

Preoperative advanced MRI offers a critical non-invasive window into GBM pathophysiology, capable of elucidating mechanisms of vascularity, immune composition, and cellular proliferation. Among the modalities reviewed, diffusion (DWI and DTI/DKI) metrics currently provide the most reproducible biological correlates, while PWI has a strong basis for clinical decision-making via vascular information despite inconsistent correlations with histology. APTw imaging and MRS show strong potential for metabolic and proliferative profiling, while QSM demonstrates robust correlations with iron-related immune markers. However, APTw and QSM require multi-centre harmonisation and prospective validation prior to being available clinically.

Collectively, these findings highlight the complementarity of advanced MRI modalities and the promise of multimodal integration for comprehensive tumour characterisation. However, translation into routine clinical practice is hindered by methodological heterogeneity and a lack of standardisation. Based on this review, recommended future directions include focusing on harmonised imaging protocols, standardised post-processing, large-scale validation studies, and the development of integrated imaging frameworks to enable personalised treatment planning, prognostication, and therapeutic monitoring in GBM.

## Figures and Tables

**Table 1:** Scoping review eligibility criteria. Abbreviations: GBM, glioblastoma; MRI, magnetic resonance imaging.

Inclusion	Exclusion
Newly diagnosed adult patients with treatment-naïve GBM	Non-standard treatment (deviation from the Stupp protocol [10])
Minimum sample size of five	Exclusive utilisation of conventional MRI techniques
Derives biological insight from MRI-based metrics	Implementation of machine learning, artificial intelligence, or radiomics
Primary sources of evidence, available in English, from peer-reviewed journals	

**Table 2:** Characteristics of individual sources of evidence (chronological order). Abbreviations: IDHwt, isocitrate dehydrogenase wildtype; TERTp, telomerase reverse transcriptase promoter; MGMTp, O6-methylguanine-DNA methyltransferase promoter; CEST, chemical exchange saturation transfer; EGFR, epidermal growth factor receptor.

Reference	Country	Study Design	Sample Size	IDHwt Status	Field Strength (Tesla)	Biological Aspect(s)	MR Modalities
Calvo-Imirizaldu et al., 2025 [42]	Spain	Prospective	17	Reported	3	Vasculature	Perfusion
Chen et al., 2025 [43]	China	Retrospective	79	Reported	3	TERTp, MGMTp	Diffusion
Kuroda et al., 2025 [44]	Japan	Retrospective	48	Reported	3	Brain microstructure, Vasculature	Diffusion, Perfusion
Ying et al., 2025 [45]	China	Prospective	37	Reported	3	Brain microstructure	CEST
Zhang et al., 2025 [46]	China	Retrospective	124	Reported	3	Immune response	Diffusion
Chida et al., 2024 [47]	Japan	Retrospective	50	Reported	3	MGMTp	Perfusion
Dono et al., 2024 [48]	United States	Retrospective	178	Reported	1.5, 3	Vasculature	Diffusion
Kamimura et al., 2024 [49]	Japan	Retrospective	114	Reported	1.5, 3	TERTp, MGMTP	Diffusion
Vikhrova et al., 2024 [50]	Russia	Prospective	40	Reported	3	Brain microstructure, EGFR, MGMTp	Diffusion, Perfusion
Wurtemberger et al., 2024 [51]	Germany	Retrospective	10	Reported	3	Brain microstructure	Diffusion
Genc et al., 2023 [52]	Turkey	Retrospective	25	Reported	3	Brain microstructure	Diffusion
Inoue et al., 2023 [53]	Japan	Prospective	12	Reported	3	Brain microstructure	CEST
Ladenhauf et al., 2023 [54]	Austria	Retrospective	42	Reported	3	MGMTp	Diffusion, Perfusion
Ohba et al., 2023 [55]	Japan	Retrospective	27	Reported	3	Brain microstructure, MGMTp	CEST
Sun et al., 2023 [56]	China	Prospective	23	Reported	3	Brain microstructure	Diffusion, Susceptibility
Zakharova et al., 2023 [57]	Russia	Prospective	37	Reported	3	Brain microstructure	Diffusion, Perfusion
Zhou et al., 2023 [58]	China	Retrospective	140	Reported	3	Immune response	Diffusion
Alvarez-Torres et al., 2022 [59]	Spain	Retrospective	17	Reported	1.5, 3	Vasculature	Perfusion
Nakamura et al., 2022 [60]	Japan	Prospective	23	Reported	3	Immune response	Spectroscopy
Nazem et al., 2022 [61]	United States	Retrospective	19	Reported	3	Immune response	Perfusion, Susceptibility
Stumpo et al., 2022 [62]	Switzerland	Prospective	6	Reported	3	Vasculature	Functional
Yuan et al., 2022 [63]	China	Prospective	13	Reported	3, 7	Brain microstructure	CEST, Spectroscopy
Liesche-Starnecker et al., 2021 [64]	Germany	Prospective	43	Reported	3	Vasculature	Diffusion, Perfusion
Stadlbauer et al., 2021 [65]	Germany	Retrospective	64	Reported	3	Tumour Microenvironment	Physiologic
Rohrich et al., 2020 [66]	Germany	Retrospective	13	Reported	3	Immune response	Diffusion, Perfusion
Rotkopf et al., 2020 [67]	Germany	Retrospective	12	Reported	3	Vasculature	Perfusion
Schon et al., 2020 [68]	Germany	Prospective	31	Reported	3	Vasculature	CEST, Perfusion
Han et al., 2018 [69]	China	Retrospective	77	Reported	3	MGMTp	Diffusion, Perfusion
Stadlbauer et al., 2018 [70]	Germany	Prospective	52	Reported	3	Tumour Microenvironment	Physiologic
Bakas et al., 2017 [71]	United States	Mixed	142	Inferred	3	EGFR	Diffusion, Perfusion
Valentini et al., 2017 [72]	Italy	Prospective	12	Reported	1.5	Brain microstructure	Diffusion, Perfusion, Spectroscopy
Barajas et al., 2015 [73]	United States	Prospective	10	Inferred	1.5	Vasculature	Perfusion
Gupta et al., 2015 [6]	United States	Retrospective	106	Inferred	1.5, 3	EGFR	Perfusion
Young et al., 2013 [74]	United States	Retrospective	147	Inferred	1.5, 3	EGFR	Diffusion
Tykocinski et al., 2012 [75]	United States	Retrospective	132	Inferred	1.5, 3	EGFR, Vasculature	Perfusion
Blasel et al., 2011 [76]	Germany	Retrospective	15	Inferred	3	Brain microstructure	Perfusion, Spectroscopy
Ulmer et al., 2009 [77]	Germany	Prospective	11	Inferred	1.5	Vasculature	Perfusion

**Table 3:** Summary of the results of the 15 eligible studies investigating the microstructure of GBM. Abbreviations: AD, axial diffusivity; ADC, apparent diffusion coefficient; APTw, amide proton transfer-weighted; CBF, cerebral blood flow; CBV, cerebral blood volume; CE, contrast-enhancing; CEST, chemical exchange saturation transfer; Cho, choline; Cr, creatine; DKI, diffusion kurtosis imaging; DWI, diffusion-weighted imaging; FA, fractional anisotropy; FU, follow-up; GBM, glioblastoma; H&E, haematoxylin and eosin; ICVF, intracellular volume fraction; IQR, interquartile range; Ki-67, protein marker of cellular proliferation; LI, labelling index; MAD, median absolute deviation; MD, mean diffusivity; MRP, magnetic resonance perfusion; MRS, magnetic resonance spectroscopy; MTT, mean transit time; NAA, N-acetylaspartate; NAWM, normal-appearing white matter; NET, non-enhancing tumour; P10, 10th percentile; P90, 90th percentile; PWI, perfusion-weighted imaging; QSM, quantitative susceptibility mapping; rCBV, relative cerebral blood volume; RD, radial diffusivity; RMS, root mean square; SS, striate sign; SWI, susceptibility-weighted imaging; tCho, total choline; V-CSF, cerebrospinal fluid volume fraction; V-ISO, isotropic volume fraction; V-intra, intracellular volume fraction.

Modality	Reference	Reference Standard	Key Findings
CEST	[53]	Ki-67	Mean APTw (27.2% ± 12.8) is higher in GBM vs. other gliomas (*p* < 0.001); APTw may localise infiltrating tumour cells. Average Ki-67 LI = 37 ± 14.
	[55]	MIB-1, p53	Moderate correlation between mean APTw signal and MIB-1 LI; no correlation with p53; percentile-based APTw metrics not predictive.
	[45]	H&E, Ki-67	Apparent_rAPT and QUASS_rAPT positively correlated with cell density (r = 0.588, *p* = 0.001; r = 0.801, *p* < 0.001) and Ki-67 (r = 0.617, *p* < 0.001; r = 0.776, *p* < 0.001). CEST@2 ppm showed no correlation. QUASS_rMT&NOE negatively correlated with cell density (r = −0.494, *p* = 0.009) and Ki-67 (r = −0.527, *p* = 0.005). QUASS algorithm improved APTw utility.
DWI	[51]	N/A	FA, microFA, V-intra, and ICVF decreased compared to NAWM (*p* < 0.05). MD, RD, AD, microADC, V-CSF, V-ISO, and ODI increased compared to NAWM (*p* < 0.05).
	[52]	N/A	Patients with right- and left-sided GBMs exhibited changes in different diffusion parameters.
PWI	[67]	Ki-67	Linear model with Ki-67 and either rCBV or wavelet-MRP showed no significant association between parameters (*p* = 0.992, *p* = 0.899, respectively). Non-linear modelling yielded similarly insignificant results (*p* = 0.62, *p* = 0.70, respectively).
CEST + MRS	[63]	N/A	Patient-wise analysis revealed no CEST-MRS correlation. Voxel-wise: CEST-MRS moderate correlation. Probability map from combined APT/MRS showed efficacy in the ability to predict tumour presence.
CEST + PWI	[68]	H&E	Significant correlation between APTw and cellularity (Spearman’s ρ = 0.37, *p* = 0.02886).No correlation between CBV and cellularity (Spearman’s ρ = 0.11, *p* = 0.52).
DWI + PWI	[50]	Ki-67	Mean CBF_max_ when Ki-67 > 20% = 160.45 ± 46.01; when Ki-67 < 20% = 111.83 ± 66.26, *p* = 0.04.
	[57]	Ki-67, CD133, Bcl-2 EA	Significant (*p* < 0.05) correlations between DKI and both Ki-67 labelling index and Bcl-2 expression activity in the highly perfused enhancing tumour core and in perifocal infiltrative oedema zone. CBF correlated with Ki-67 LI in highly perfused enhancing tumour core.
	[64]	H&E	No significant correlation between CBV and cellularity (ρ = 0.129, *p* = 0.106).MD and FA were negatively associated with cellularity (MD: ρ = −0.154, *p* = 0.50; FA: ρ = −0.095, *p* = 0.231).
	[44]	H&E, Ki-67, CD31	Ki-67 and cell density were higher in NETs than in oedema (*p* < 0.05 and *p* < 0.01, respectively). Ki-67 significant higher in CE than in NETs (*p* < 0.05). Cell density between CE and NET was comparable (*p* > 0.05). CBF ratio showed a correlation with cell density (R = 0.400, *p* = 0.023) and Ki-67 index (R = 0.374, *p* = 0.034). The MTT ratio also showed a correlation with cell density (R = 0.409, *p* = 0.02) and Ki-67 index (R = 0.322, *p* = 0.0003). No correlation between CBV ratio and cell density or Ki-67.
DWI + SWI	[56]	Ki-67	Positive correlations between the Ki-67 LI and QSM histogram features: P90, IQR, maximum, MAD, RMS, skewness, and variance (ρ ranged from 0.407 to 0.531, *p* < 0.01 for all). Negative correlations with Ki-67 LI were found for P10 of QSM (ρ = −0.452, *p* = 0.001) and P10 of ADC (ρ = −0.554, *p* < 0.001).
DWI + PWI + MRS	[72]	Ki-67, MIB-1	The Ki-67/MIB-1 LI was significantly associated with the Cho/Cr (r = 0.95, *p* = 0.03). In the CE region, rCBV, Cho/Cr, and Cho/NAA values corresponded to the highest Ki-67/MIB-1 LI.
MRS + PWI	[76]	N/A	Increased tCho concentrations within the SS showing de novo CE at FU.

**Table 4:** Summary of the results of the ten eligible studies investigating the vasculature of GBM. Abbreviations: ADC, apparent diffusion coefficient; BOLD, blood oxygen level-dependent; CBF, cerebral blood flow; CBV, cerebral blood volume; CE, contrast-enhancing; CVR, cerebrovascular reactivity; DSC, dynamic susceptibility contrast; DWI, diffusion-weighted imaging; FA, fractional anisotropy; FDR, false discovery rate; GBM, glioblastoma; GM, grey matter; H&E, haematoxylin and eosin; MRP, magnetic resonance perfusion; MTT, mean transit time; MVA, micro-vessel area; NET, non-enhancing tumour; NGS, next-generation sequencing; PH, peak height; PSR, percentage signal recovery; PWI, perfusion-weighted imaging; rCBF, relative cerebral blood flow; rCBV, relative cerebral blood volume; ROI, region of interest; rTBV, relative tumour blood volume; VEGF-A, vascular endothelial growth factor A; VOI, volume of interest.

Modality	Reference	Reference Standard	Key Findings
DWI	[48]	NGS	Diffusion patterns differed significantly between amplified vs. non-amplified GBMs (*p* = 0.00007; FDR-adjusted *p* = 0.002); amplified GBMs mostly non-restricting/mixed diffusion; non-amplified GBMs often restricting.
PWI	[42]	N/A	Tumour VOI had lower CVR than contralateral GM (*p* = 0.0016); strong inverse correlation between baseline CBF and CVR (ρ = −0.71, *p* < 0.001); ipsilateral GM CVR lower than contralateral GM, suggesting impaired autoregulation.
	[59]	H&E	Higher rCBV regions had significantly larger MVA (rCBV_mean_ ρ = 0.38, *p* = 0.0008; rCBV_max_ ρ = 0.42, *p* < 0.0002); significant differences in rCBV between ROIs with vs. without microvessels (*p* < 0.0016).
	[67]	CD31 expression	Wavelet-MRP significantly associated with CD31 expression (*p* = 0.043); rCBV not associated with CD31 (*p* = 0.297); wavelet-MRP predictive of vessel density; strong linear correlation between wavelet-MRP and rCBV (R = 0.81).
	[73]	Gene expression	25 pro-angiogenic genes differentially expressed between CE and NET regions; VEGF-A expression correlated with DSC metrics (CBV, PH, PSR) (*p* < 0.05); VEGF-A-T2 correlated with rCBV (r = 0.42, *p* = 0.03) and PSR (r = −0.42, *p* = 0.03).
	[75]	VEGF expression	rTBV not associated with lnVEGF (F-ratio = 2.71, *p* = 0.102).
	[77]	N/A	Tumour rCBV and rCBF significantly higher than adjacent GM (*p* < 0.0001); strong correlation between tumour and adjacent GM perfusion (rCBF R = 0.69; rCBV R = 0.85); increased tumour perfusion does not reduce perfusion in surrounding tissue.
DWI + PWI	[44]	H&E, histology (MVA)	MVA higher in NET vs. oedema (*p* < 0.05) and CE tumour vs. NET (*p* < 0.05); CBF ratio correlated with MVA (R = 0.443, *p* = 0.011); MTT ratio correlated with MVA (R = 0.430, *p* = 0.014); ADC ratio higher in NET vs. oedema (*p* < 0.05).
	[64]	H&E	CBV increased with neovascularisation (*p* = 0.003); FA tended to be lower in highly vascular regions (*p* = 0.215).
Functional	[62]	N/A	No differences using binarised hypercapnic or hypoxic maps; hyperoxic maps showed a trend toward differential %BOLD signal change between positive and negative voxels during other stimuli. Negative voxels during hyperoxia had opposite and greater magnitude change during hypercapnia (−0.055 vs. 0.012). Two patients showed only negative %BOLD during hypoxia and positive during hyperoxia; others showed negative %BOLD near tumour zones during hyperoxia. Suggests GBM affects whole-brain vascular/metabolic response.

**Table 5:** Summary of the results of the six eligible studies investigating MGMTp methylation in GBM. Abbreviations: ADC, apparent diffusion coefficient; APTw, amide proton transfer-weighted; CE, contrast-enhancing; CEST, chemical exchange saturation transfer; DWI, diffusion-weighted imaging; GBM, glioblastoma; MGMTp, O6-methylguanine-DNA methyltransferase promoter; PCR, polymerase chain reaction; PWI, perfusion-weighted imaging; ROI, region of interest; rCBF, relative cerebral blood flow; rCBV, relative cerebral blood volume; rMTT, relative mean transit time.

Modality	Reference	Reference Standard	Key Findings
CEST	[55]	PCR	No correlation between MGMTp status and APTw predictable diagnosis; no significant differences in mean, 1st/100th percentile, or width1–100 APTw signals between methylated and unmethylated GBM.
DWI	[43]	PCR	ADC_min_ significantly lower in unmethylated MGMTp subgroup vs. methylated (*p* = 0.005); no other histogram features differed.
PWI	[47]	PCR	rCBV significantly higher in methylated MGMTp tumours (*p* < 0.005); rCBF also higher in methylated group (*p* < 0.05); rMTT showed no difference. Suggests rCBV and rCBF as potential preoperative predictors.
DWI + PWI	[54]	PCR	ADC values in ROI-3 (adjacent to CE region) higher in unmethylated MGMTp group (*p* = 0.002); effect more pronounced after normalisation (*p* = 0.0007); no other ROIs showed significant differences.
	[69]	N/A	ADC lower in unmethylated MGMTp group vs. methylated (*p* < 0.001); rCBF higher in unmethylated group (*p* < 0.001).
	[50]	N/A	Significant difference only for mean ADC_min_ between methylated and unmethylated groups (*p* = 0.02).

**Table 6:** Summary of the results of the five eligible studies investigating EGFR subtypes of GBM. Abbreviations: ADC, apparent diffusion coefficient; CBF, cerebral blood flow; CE, contrast-enhancing; DSC, dynamic susceptibility contrast; DTI, diffusion tensor imaging; DWI, diffusion-weighted imaging; EGFR, epidermal growth factor receptor; EGFRvIII, epidermal growth factor receptor variant III; FDR, false discovery rate; GBM, glioblastoma; NGS, next-generation sequencing; OR, odds ratio; PCR, polymerase chain reaction; PHI, peritumoral heterogeneity index; PSR, percentage signal recovery; PWI, perfusion-weighted imaging; rCBV, relative cerebral blood volume; rPH, relative peak height; rTBV, relative tumour blood volume.

Modality	Reference	Reference Standard	Key Findings
DWI	[74]	Interphase fluorescence in situ hybridisation	Lower ADC values in EGFR-amplified tumours (*p* < 0.01 for all ADC metrics); strongest correlations for ADCROI (CE tumour with minimum ADC) (*p* = 0.0003) and mean ADC of CE region (*p* = 0.0007).
PWI	[6]	Interphase fluorescence in situ hybridisation.PCR	Higher EGFR amplification associated with higher rCBVmedian (FDR *p* = 0.03) and lower PSR (FDR *p* = 0.052); rPH not significant (*p* = 0.30). EGFRvIII mutation showed no significant correlation with perfusion metrics (*p* > 0.19).
	[75]	PCR	EGFRvIII-expressing GBMs had significantly higher rTBV in CE region (1.5 T: *p* = 0.001; 3 T: *p* = 0.000); rTBV is independent predictor of EGFRvIII expression (McFadden’s ρ^2^ = 0.23, *p* = 0.000; OR = 2.70). EGFR wildtype cannot be predicted using rTBV.
DWI + PWI	[50]	N/A	No significant correlation between ADC or CBF metrics and EGFR status.
	[71]	In-house NGS-based assay (validated with detection by Taqman Reverse Transcription-PCR).	PHI derived from DSC metrics significantly discriminated EGFRvIII mutation status (*p* = 4.0033 × 10^−10^); PHI from DTI metrics showed no discriminatory ability.

**Table 7:** Summary of the results of the two eligible studies investigating TERTp mutations in GBM. Abbreviations: ADC, apparent diffusion coefficient; CE, contrast-enhancing; DWI, diffusion-weighted imaging; GBM, glioblastoma; NET, non-enhancing tumour; NGS, next-generation sequencing; P10, 10th percentile; PCR, polymerase chain reaction; TERTp, telomerase reverse transcriptase promoter; TERTp-mt, TERTp-mutated; TERTp-wt, TERTp wildtype.

Modality	Reference	Reference Standard	Key Findings
DWI	[49]	PCR	TERTp-mutated GBMs had significantly lower ADC_min_ in NET vs. TERTp-wt (*p* < 0.01); no difference in CE region ADC. Histology confirmed NET with low ADC had aggressive invasion; sparse tumour cells in NET with high ADC. Suggests TERTp-mt GBMs are more invasive in NET.
	[43]	NGS	Significant differences between TERTp-mt and TERTp-wt in ADC_mean_ (*p* = 0.003), ADC_min_ (*p* = 0.019), ADC_p10_ (*p* = 0.007); entropy higher in TERTp-mt group (*p* < 0.001).

**Table 8:** Summary of the results of the five eligible studies investigating the immune response in GBM. Abbreviations: ADC, apparent diffusion coefficient; CE, contrast-enhancing; Cr, creatine; DWI, diffusion-weighted imaging; FAP, fibroblast activation protein; GBM, glioblastoma; Glu, glutamate; GSCs, glioma stem cells; H&E, haematoxylin and eosin; IHC, immunohistochemistry; Lac, lactate; MRS, magnetic resonance spectroscopy; NAA, N-acetylaspartate; NET, non-enhancing tumour; OS, overall survival; P/C ratio, periphery-to-core ratio; PD-L1, programmed death-ligand 1; PET/CT, positron emission tomography/computed tomography; PWI, perfusion-weighted imaging; QSM, quantitative susceptibility mapping; rCBV, relative cerebral blood volume; RT–qPCR, reverse transcription quantitative polymerase chain reaction; SWI, susceptibility-weighted imaging; TAMs, tumour-associated macrophages; TME, tumour microenvironment; VASARI, Visually AcceSSible Rembrandt Images.

Modality	Reference	Reference Standard	Key Findings
DWI	[58]	Anti-CD163 antibody and anti-CD68 antibody.	CD163+ macrophage infiltration showed correlation with ADC_mean_ in CE region (r = 0.208, *p* = 0.014); ADC_min_ showed no correlation. Suggests restricted diffusion with increased proliferation.
	[46]	PD-L1 expression via IHC	VASARI feature F5 (enhancing tumour proportion) higher in low PD-L1 group (*p* = 0.003); other features and diffusion patterns showed no significant differences (*p* > 0.05). PD-L1 negatively correlated with OS but not diffusion properties.
MRS	[60]	RT–qPCR	Highly invasive GBMs had higher CD44 P/C ratio (*p* = 0.027); seizure subgroups showed higher Glu/Cr and NAA/Cr (*p* = 0.011, *p* = 0.007); type-C (post-treatment seizures) had highest Glu/Cr, NAA/Cr, Lac/Cr vs. type-A (*p* ≤ 0.037). CD44 expression higher in type-C vs. type-B (*p* < 0.05). Suggests CD44 on GSCs linked to recurrence and seizures.
DWI + PWI	[66]	FAP-specific PET/CT	Moderate positive correlation between FAP-PET signal and rCBV in NET (r = 0.229); smaller effect in CE region (r = 0.09); no correlation with ADC in NET or CE. Supports FAP-PET as complementary to MRI.
PWI + SWI	[61]	H&E, CD68, CD86, CD206, l-Ferritin.	QSM mean susceptibility in CE region correlated with l-ferritin (r = 0.56, *p* = 0.007), CD68 (ρ = 0.52, *p* = 0.034), CD86 (ρ = 0.7, *p* = 0.001); no correlation with CD206. Combined CE + necrotic susceptibility correlated with l-ferritin (r = 0.72, *p* = 0.001) and CD86 (r = 0.63, *p* = 0.005). Suggests M1 TAMs store iron, M2 TAMs release iron into TME.

**Table 9:** Summary of the results of the two eligible studies investigating the tumour microenvironment in GBM. Abbreviations: GBM, glioblastoma; OxPhos, oxidative phosphorylation; TME, tumour microenvironment.

Modality	Reference	Reference Standard	Key Findings
Physiologic	[70]	N/A	Two GBM phenotypes identified: (1) glycolysis-dominant with functional neovascularisation; (2) necrosis/hypoxia-dominant with defective neovascularisation. Volumes of all TMEs (except OxPhos + neovascularisation) differed significantly (*p* < 0.005). Enables non-invasive detection of tumour-supportive niches.
	[65]	N/A	Vital tumour (OxPhos + aerobic glycolysis) comprised ~54% ± 24% of GBM TME; aerobic glycolysis 37% ± 22%, OxPhos 17% ± 6%, necrosis 22% ± 11%, hypoxia with neovascularisation 15% ± 10%, hypoxia without neovascularisation 9% ± 7% (total hypoxia 24% ± 16%). Two-thirds of vital tumour energy production via aerobic glycolysis.

## Data Availability

The original contributions presented in this study are included in the article/Supplementary Material. Further inquiries can be directed to the corresponding authors.

## Supplementary Materials

The following supporting information can be downloaded at: https://www.mdpi.com/article/10.3390/cancers18040645/s1, Table S1: Detailed, database-specific search strategies; Table S2: Data extraction matrix for eligible articles.

## Abbreviations

The following abbreviations are used in this manuscript:

11C-MET-PET	Carbon-11 Methionine Positron Emission Tomography
18F-FDG PET	Fluorine-18 Fluorodeoxyglucose Positron Emission Tomography
AD	Axial Diffusivity
ADC	Apparent Diffusion Coefficient
AI	Artificial Intelligence
AK	Axial Kurtosis
APTw	Amide Proton Transfer-Weighted
ASL	Arterial Spin Labelling
AWF	Axonal Water Fraction
AxEAD	Axial Extra-Axonal Diffusivity
AxIAD	Axial Intra-Axonal Diffusivity
BOLD	Blood Oxygen Level Dependent
CBF	Cerebral Blood Flow
CBV	Cerebral Blood Volume
CE	Contrast-Enhanced
CEST	Chemical Exchange Saturation Transfer
Cho	Choline
CMRO_2_	Cerebral Metabolic Rate of Oxygen
CNR	Choline-to-N-Acetylaspartate Ratio
Cr	Creatine
CT	Computed Tomography
CVR	Cerebrovascular Reactivity
DKI	Diffusion Kurtosis Imaging
DSC	Dynamic Susceptibility Contrast
DTI	Diffusion Tensor Imaging
DWI	Diffusion-Weighted Imaging
EA	Extra-Axonal
EGFR	Epidermal Growth Factor Receptor
EGFRvIII	Epidermal Growth Factor Receptor Variant III
FA	Fractional Anisotropy
FAP	Fibroblast Activation Protein
FDR	False Discovery Rate
FET-PET	Fluoroethyl-L-tyrosine Positron Emission Tomography
FGF	Fibroblast Growth Factor
FLAIR	Fluid-Attenuated Inversion Recovery
FU	Follow-Up
GBM	Glioblastoma
Glu	Glutamate
GM	Grey Matter
GSC	Glioma Stem Cells
H&E	Haematoxylin and Eosin
HIF	Hypoxia-Inducible Factor
ICVF	Intracellular Volume Fraction
IDH	Isocitrate Dehydrogenase
IDHmt	IDH Mutant
IDHwt	IDH Wildtype
IHC	Immunohistochemistry
IQR	Interquartile Range
JBI	Joanna Briggs Institute
KA	Kurtosis Anisotropy
Lac	Lactate
LI	Labelling Index
MAD	Median Absolute Deviation
MD	Mean Diffusivity
MGMTp	O6-Methylguanine-DNA Methyltransferase Promoter
microADC	Microscopic Apparent Diffusion Coefficient
microFA	Microscopic Fractional Anisotropy
mitoPO_2_	Mitochondrial Partial Pressure of Oxygen
MK	Mean Kurtosis
ML	Machine Learning
MR	Magnetic Resonance
MRI	Magnetic Resonance Imaging
MRP	Magnetic Resonance Perfusion
MRS	Magnetic Resonance Spectroscopy
MRSI	Magnetic Resonance Spectroscopic Imaging
MT&NOE	Magnetisation Transfer and Nuclear Overhauser Effect
MTT	Mean Transit Time
MVA	Micro-Vessel Area
NAA	N-Acetylaspartate
NAGM	Normal-Appearing Grey Matter
NAWM	Normal-Appearing White Matter
NET	Non-Enhancing Tumour
NGS	Next-Generation Sequencing
ODI	Orientation Dispersion Index
OEF	Oxygen Extraction Fraction
OR	Odds Ratio
OS	Overall Survival
OxPhos	Oxidative Phosphorylation
P/C	Periphery-to-Core Ratio
P10	10th Percentile
P90	90th Percentile
pcASL	Pseudo-Continuous Arterial Spin Labelling
PCNSL	Primary Central Nervous System Lymphoma
PCR	Polymerase Chain Reaction
PDGF	Platelet-Derived Growth Factor
PD-L1	Programmed Death-Ligand 1
PET/CT	Positron Emission Tomography / Computed Tomography
PFS	Progression-Free Survival
PH	Peak Height
PHI	Peritumoral Heterogeneity Index
PRISMA-ScR	Preferred Reporting Items for Systematic Reviews and Meta-Analyses Extension for Scoping Reviews
PSR	Percentage Signal Recovery
pv-oedema	Purely Vasogenic Oedema
PWI	Perfusion-Weighted Imaging
QSM	Quantitative Susceptibility Mapping
QUASS	Quasi-Steady-State
RadEAD	Radial Extra-Axonal Diffusivity
RadIAD	Radial Intra-Axonal Diffusivity
rCBF	Relative Cerebral Blood Flow
rCBV	Relative Cerebral Blood Volume
RD	Radial Diffusivity
RK	Radial Kurtosis
RMS	Root Mean Square
rMTT	Relative Mean Transit Time
ROI	Region of Interest
rPH	Relative Peak Height
RQ	Research Question
rTBV	Relative Tumour Blood Volume
RT-qPCR	Reverse Transcription Quantitative Polymerase Chain Reaction
SS	Striate Sign
SWI	Susceptibility-Weighted Imaging
T1w	T1-weighted
TAM	Tumour-Associated Macrophages
tCho	Total Choline
TERTp	Telomerase Reverse Transcriptase Promoter
TERTp-mt	TERTp Mutant
TERTp-wt	TERTp Wildtype
TME	Tumour Microenvironment
TMZ	Temozolomide
TORT	Tortuosity
VAM	Vascular Architecture Mapping
VASARI	Visually AcceSSible Rembrandt Images
V-CSF	Cerebrospinal Fluid Volume
VEGF-A	Vascular Endothelial Growth Factor A
V-intra	Intracellular Volume
V-ISO	Isotropic Volume
VOI	Volume of Interest
WHO	World Health Organization
WM	White Matter
