# Supplementary material for: Exploring the Role of Advanced MRI in Understanding Glioblastoma Biology: A Scoping Review
Source: Cancers (Basel). 2026 Feb 16;18(4):645. doi: 10.3390/cancers18040645 (PMC12939606; doi:10.3390/cancers18040645)
Supplement: Supplementary file 1 [file cancers-18-00645-s001.zip › Cancers Scoping Review Supplementary Materials.pdf]

Supplementary Materials

Table S1. Detailed, database-specific search strategies.

Database	Search Strategy
PubMed	(MR[Title] OR MRI[Title/Abstract] OR "magnetic resonance"[Title/Abstract] OR "APT" OR "CEST" OR "amide proton transfer" OR "chemical exchange saturation transfer" OR "DWI" OR "DTI" OR "diffusion-weighted imag*" OR "diffusion tensor imag*" OR "SWI" OR "QSM" OR "susceptibility-weighted imag*" OR "quantitative susceptibility map*" OR "dynamic contrast enhanc*" OR "dynamic susceptibility contrast-enhanc*" OR "DKI" OR "diffusion kurtosis imag*" OR "PWI" OR "perfusion-weighted imag*" OR "arterial spin label*") AND (glioblastoma[Title/Abstract] OR GBM[Title/Abstract] OR "HGG" OR ((glioma[Title/Abstract] OR astrocytoma[Title/Abstract] OR "brain tumo*"[Title/Abstract]) AND (("grade 4"[Title/Abstract:~8]) OR ("grade IV"[Title/Abstract:~8]) OR "high-grade"[Title/Abstract:~4]))) NOT ((review[pt]) OR (systematic review[pt]) OR (meta-analysis[pt]) OR (case reports[pt]))
Scopus	(TITLE-ABS-KEY (MRI) OR TITLE-ABS-KEY ("magnetic resonance") OR TITLE ("mr") OR TITLE-ABS-KEY ("amide-proton transfer") OR TITLE-ABS-KEY ("chemical exchange saturation transfer") OR TITLE-ABS-KEY ("diffusion-weighted imaging") OR TITLE-ABS-KEY ("diffusion tensor imaging") OR TITLE-ABS-KEY ("susceptibility-weighted imaging") OR TITLE-ABS-KEY ("quantitative susceptibility mapping") OR TITLE-ABS-KEY ("arterial spin labelling") OR TITLE-ABS-KEY ("diffusion kurtosis imaging") OR TITLE-ABS-KEY ("perfusion-weighted imaging")) AND (TITLE-ABS-KEY (glioblastoma) OR TITLE-ABS-KEY (GBM) OR TITLE-ABS-KEY ("high-grade glioma")) AND (SUBJAREA(MEDI) OR SUBJAREA(BIOC) OR SUBJAREA(NEUR) OR SUBJAREA(HEAL) OR SUBJAREA(CHEM) OR SUBJAREA(PHYS) OR SUBJAREA(PHAR) OR SUBJAREA(IMMU) OR SUBJAREA(AGRI)) AND (KEY (adult) OR KEY (human) OR KEY(humans) OR KEY("in vivo study")) AND NOT KEY ("human tissue") AND NOT KEY ("case report") AND NOT KEY ("child") AND NOT KEY ("nonhuman") AND NOT KEY ("animals") AND NOT KEY ("animal experiment") AND NOT KEY ("animal model") AND NOT KEY ("mouse") AND NOT KEY ("animal") AND NOT KEY ("cell line, tumor") AND NOT KEY ("animal tissue") AND NOT KEY ("child, preschool") AND NOT KEY ("preschool child") AND (LIMIT-TO (SRCTYPE, "j")) AND (LIMIT-TO (LANGUAGE ,"English")) AND (LIMIT-TO (DOCTYPE, "ar"))
Cochrane	Title Abstract Keyword ("magnetic resonance" OR MRI OR MR OR "amide proton transfer" OR "chemical exchange saturation transfer" OR "diffusion-weighted imaging" OR "diffusion tensor imaging" OR "susceptibility-weighted imaging" OR "quantitative susceptibility mapping" OR "diffusion kurtosis imaging" OR "perfusion-weighted imaging" OR "arterial spin labelling") AND Title Abstract Keyword (glioblastoma OR GBM OR "high-grade glioma")
EBSCO (Academic Search Ultimate)	AB (("magnetic resonance" OR MRI OR MR OR "amide proton transfer" OR "chemical exchange saturation transfer" OR "diffusion-weighted ima*" OR "diffusion tensor ima*" OR "susceptibility-weighted ima*" OR "quantitative susceptibility map*" OR "diffusion kurtosis ima*" OR "perfusion-weighted ima*" OR "arterial spin labe*") AND (glioblastoma OR GBM OR "high-grade glioma")) OR KW (("magnetic resonance" OR MRI OR MR OR "amide proton transfer" OR "chemical exchange saturation transfer" OR "diffusion-weighted ima*" OR "diffusion tensor ima*" OR "susceptibility-weighted ima*" OR "quantitative susceptibility map*" OR "diffusion kurtosis ima*" OR "perfusion-weighted ima*" OR "arterial spin labe*") AND (glioblastoma OR GBM OR "high-grade glioma"))

Embase (Ovid)	Abstract ("magnetic resonance" OR MRI OR "amide proton transfer" OR "chemical exchange saturation transfer" OR "diffusion-weighted imag*" OR "diffusion tensor imag*" OR "susceptibility-weighted imag*" OR "quantitative susceptibility map*" OR "diffusion kurtosis imag*" OR "perfusion-weighted imag*" OR "arterial spin label*") AND (glioblastoma OR GBM OR "high-grade glioma") OR Title ("magnetic resonance" OR MRI OR MR OR "amide proton transfer" OR "chemical exchange saturation transfer" OR "diffusion-weighted imag*" OR "diffusion tensor imag*" OR "susceptibility-weighted imag*" OR "quantitative susceptibility map*" OR "diffusion kurtosis imag*" OR "perfusion-weighted imag*" OR "arterial spin label*") AND (glioblastoma OR GBM OR "high-grade glioma") OR Keyword Heading ("magnetic resonance" OR MRI OR MR OR "amide proton transfer" OR "chemical exchange saturation transfer" OR "diffusion-weighted imag*" OR "diffusion tensor imag*" OR "susceptibility-weighted imag*" OR "quantitative susceptibility map*" OR "diffusion kurtosis imag*" OR "perfusion-weighted imag*" OR "arterial spin label*") AND (glioblastoma OR GBM OR "high-grade glioma")

Table S2. Data extraction matrix for eligible articles. Abbreviations: MRI, magnetic resonance imaging; B0, static magnetic field strength; TR, repetition time; TE, echo time.

Year	First Author	Title	Country	MRI Modality	MRI Technique	MRI Sequences	MRI Metrics	B0 / TR / TE	Post-Processing Method	Analysis Method	Clinical Aspect	Relation to Biology	Limitations	Suggestions	Known Mutations	Sample Size	Sex / Mean Age	Study Design	Aims	Key Points
